# Transplantation of Immortalized CD34+ and CD34- Adipose-Derived Stem Cells Improve Cardiac Function and Mitigate Systemic Pro-Inflammatory Responses

**DOI:** 10.1371/journal.pone.0147853

**Published:** 2016-02-03

**Authors:** Jong-Ho Kim, Seung-Cheol Choi, Chi-Yeon Park, Jae-Hyoung Park, Ji-Hyun Choi, Hyung-Joon Joo, Soon-Jun Hong, Do-Sun Lim

**Affiliations:** Department of Cardiology, Cardiovascular Center, Korea University Anam Hospital, Seoul, Republic of Korea; French Blood Institute, FRANCE

## Abstract

Adipose-derived stem cells (ADSCs) have the potential to differentiate into various cell lineages and they are easily obtainable from patients, which makes them a promising candidate for cell therapy. However, a drawback is their limited life span during *in vitro* culture. Therefore, hTERT-immortalized CD34+ and CD34- mouse ADSC lines (mADSCs^hTERT^) tagged with GFP were established. We evaluated the proliferation capacity, multi-differentiation potential, and secretory profiles of CD34+ and CD34- mADSCs^hTERT^ *in vitro*, as well as their effects on cardiac function and systemic inflammation following transplantation into a rat model of acute myocardial infarction (AMI) to assess whether these cells could be used as a novel cell source for regeneration therapy in the cardiovascular field. CD34+ and CD34- mADSCs^hTERT^ demonstrated phenotypic characteristics and multi-differentiation potentials similar to those of primary mADSCs. CD34+ mADSCs^hTERT^ exhibited a higher proliferation ability compared to CD34- mADSCs^hTERT^, whereas CD34- mADSCs^hTERT^ showed a higher osteogenic differentiation potential compared to CD34+ mADSCs^hTERT^. Primary mADSCs, CD34+, and CD34- mADSCs^hTERT^ primarily secreted EGF, TGF-β1, IGF-1, IGF-2, MCP-1, and HGFR. CD34+ mADSCs^hTERT^ had higher secretion of VEGF and SDF-1 compared to CD34- mADSCs^hTERT^. IL-6 secretion was severely reduced in both CD34+ and CD34- mADSCs^hTERT^ compared to primary mADSCs. Transplantation of CD34+ and CD34- mADSCs^hTERT^ significantly improved the left ventricular ejection fraction and reduced infarct size compared to AMI-induced rats after 28 days. At 28 days after transplantation, engraftment of CD34+ and CD34- mADSCs^hTERT^ was confirmed by positive Y chromosome staining, and differentiation of CD34+ and CD34- mADSCs^hTERT^ into endothelial cells was found in the infarcted myocardium. Significant decreases were observed in circulating IL-6 levels in CD34+ and CD34- mADSCs^hTERT^ groups compared to the AMI-induced control group. Transplantation of CD34- mADSCs^hTERT^ significantly reduced circulating MCP-1 levels compared to the AMI control and CD34+ mADSCs^hTERT^ groups. GFP-tagged CD34+ and CD34- mADSCs^hTERT^ are valuable resources for cell differentiation studies *in vitro* as well as for regeneration therapy *in vivo*.

## Introduction

Adipose tissue contains a stromal vascular fraction (SVF) that is a rich source of adipose-derived stem cells (ADSCs) [1]. ADSCs can differentiate into vascular endothelial cells, vascular smooth muscle cells, and cardiomyocytes *in vitro* and *in vivo* [2]. Animal studies have demonstrated the efficacy of ADSC transplantation in the treatment of acute myocardial infarction (AMI), ischemic cardiomyopathy, dilated cardiomyopathy, and hindlimb ischemia via primarily paracrine mechanisms [2–6]. It was also known that ADSCs have anti-inflammatory as well as immunosuppressive activities via secretion of inflammatory factors including interferon gamma (IFN-γ), interleukin 1 receptor antagonist (IL-1Ra), IL-6, IL-8, IL-10, prostaglandin E2, transforming growth factor beta 1 (TGF-β1), indoleamine 2,3-dioxygenase, and nitric oxide [7–9].

Although CD34 was first identified as a hematopoietic stem cell marker, it has also been recognized as a common marker for diverse progenitors [10]. Previous studies reported that freshly isolated ADSCs were highly positive for CD34; however, ADSC expression of CD34 was rapidly downregulated during extended culture [1, 3, 11, 12]. Miranville et al. [13] demonstrated that CD34+ human ADSCs (hADSCs) could differentiate into endothelial cells, and that intravenous injection of CD34+ hADSCs into mouse ischemic hindlimb increased blood flow and the capillary density, and resulted in the incorporation of the cells into the leg vasculature. Traktuev et al. [11] also demonstrated that the majority of CD34+ hADSCs are resident pericytes that play a role in vascular stabilization. However, little is known about the functional roles of CD34 in proliferation and differentiation processes of ADSCs and the regeneration potential of infarcted myocardium.

Although ADSCs are a promising candidate for cell therapy in the cardiovascular field, they have a limited life span during *in vitro* culture. Furthermore, primary ADSCs consisting of heterogeneous cell populations hamper studies on the molecular mechanism(s) underlying the regulation of cell differentiation or proliferation, as well as studies on cell transplantation that require a genetically homogenous cell population and a sufficient number of cells.

Telomerase comprises both telomerase reverse transcriptase (TERT) and the telomerase RNA, and adds telomere repeats to chromosome ends [14]. It has been shown that adult stem cells circumvent cellular senescence by expressing TERT [15, 16]. Viral genes such as v-myc or SV40 large T-antigen have also been used to immortalize multiple cells [17, 18]. However, viral gene-transduced cell lines frequently contained viral oncogenic DNA and accompanied major cytogenic alterations [19].

Therefore, we established CD34+ and CD34- mouse ADSCs (mADSCs) by transduction with the human TERT (hTERT) gene, which is normally expressed in the human chromosome and serves a critical role in stem cell function and tissue homeostasis. For the potential applications of these cells as a novel cell source for regeneration therapy in the cardiovascular field, we also investigated differences between hTERT-immortalized CD34+ mADSCs and CD34- mADSCs, including cell surface markers, secretory profiles, proliferation and differentiation potentials *in vitro*, as well as inflammatory responses and regenerative potential after transplantation into infarcted myocardium.

## Materials and Methods

### Animal care

All procedures were approved by the Korea University Institutional Ethics Committee for animal research (KUIACUC-2014-31). Animals were strictly handled in accordance with the Guidelines for Animal Care and Use of the Korea University School of Medicine.

### Isolation and culture of primary mADSCs

mADSCs were isolated from 6 week-old male ICR mice (Hanlim Experimental Animal Laboratory, Seoul, Korea). The mice were given an intraperitoneal (IP) injection anesthesia with a mixture of ketamine (44 mg/kg; Yuhan, Gunpo, Korea) and xylazine hydrochloride (0.75 mg/kg; Bayer AB, Leverkusen, Germany), and euthanized. Mouse inguinal fat pads were dissected, minced, and digested with 0.1% Type I collagenase (Worthington Biochemical, Freehold, NJ, USA) for 45 mins at 37°C with shaking at 200 rpm. An equal volume of culture medium containing 10% fetal bovine serum (FBS; Invitrogen, Carlsbad, CA, USA) was added, followed by filtration through 100 μm nylon mesh (BD Biosciences, San Jose, CA, USA). Then, PBS washing by centrifugation at 600 × g was carried out and the cells were suspended and incubated in 160 mM NH_4_Cl for 10 mins to remove contaminating red blood cells. The cells were then plated in 100-mm tissue culture dishes following washing with PBS and were maintained in Messencult basal medium, supplemented with MSC stimulatory supplements (StemCell Technologies, Seattle, WA, USA) and 100 unit/mL penicillin and 100 μg/mL streptomycin (P/S; Invitrogen) at 37°C in a humidified incubator with 5% CO_2_. The culture media were changed every 2 days. Primary mADSCs were defined as ‘passage 1’ and were passaged at a ratio of 1:3 when they reached confluence.

### Production of retroviruses encoding hTERT-IRES-EGFP in 293GPG cells

In order to generate the pLPCX-hTERT-IRES-EGFP vector, the BglII-SalI fragment containing hTERT cDNA was polymerase chain reaction (PCR) amplified by using the pCI-neo-hEST2 (Addgene, Cambridge, MA, USA) as a template, with a sense primer containing the BglII site (underlined) (5'-ATATATAGATCTAGGCTAGCCTCGAGAATTC-3') and antisense primer containing the SalI site (underlined) (5'-ATATATGTCGACTCAGTCCAGGATGGTCTTGAAGT-3'). The BglII-SalI-digested PCR fragment containing hTERT cDNA was inserted into the BglII-SalI site of the pIRES2-EGFP vector (Clontech Laboratories, Mountain View, CA, USA). Next, the BglII-ClaI fragment containing hTERT-IRES-EGFP cDNA was PCR-amplified by using the pIRES2-EGFP vector harboring hTERT cDNA as a template with a sense primer including the BglII site (underlined) (5'-ATATATAGATCTAGGCTAGCCTCGAGAATTC-3') and antisense primer containing the ClaI site (underlined) (5'-ATATATATCGATTTACTTGTACAGCTCGTCCA-3'). Finally, the BglII-ClaI digested PCR fragment containing hTERT-IRES-EGFP cDNA was inserted into the BglII-ClaI site of pLPCX vector (Clontech Laboratories). Correct construction of the pLPCX-hTERT-IRES-EGFP was confirmed by digestion with either BglII or ClaI and both BglII and ClaI, and finally by DNA sequencing.

The 293GPG packaging cell line [20] was maintained in 293GPG medium [Dulbecco’s Modified Eagle’s Medium (DMEM) with high glucose, L-glutamine, and sodium pyruvate supplemented with 10% FBS, 1 μg/mL tetracycline, 2 μg/mL puromycin, 300 μg/mL G418, and 100 μL P/S], as previously described [20]. To produce retroviruses, 293GPG cells were plated at 1 × 10^6^ cells/mL in 60-mm tissue culture dishes in 293GPG medium and transfected with 8 μg of the pLPCX-TERT-IRES-EGFP at 80% confluence using Lipofectamine 2000 (Invitrogen) in accordance with the manufacturer's instructions. Twenty-four hrs after transfection, the culture medium was replaced with 293GPG medium without tetracycline, puromycin, or G418. Viral supernatant was collected at 48, 72, and 96 hrs. GFP expression was investigated in 293GPG cells at 48 hrs post-transfection to evaluate transfection efficiency. Supernatants from each time point were pooled, filtered through a 0.45 μm pore size filter (Thermo Fisher Scientific, Rochester, NY, USA), and stored as single-use aliquots at -80°C.

### Generation of hTERT-immortalized mADSC lines

mADSCs (passage 1) were plated at 1 × 10^5^ cells in 100-mm culture dishes in DMEM-low glucose (LG) supplemented with 10% FBS and P/S (all from Invitrogen), and infected with retroviruses harboring the pLPCX-TERT-IRES-EGFP at 60% confluence for 3 days. The cells were selected in the medium against 0.5 μg/mL puromycin during a 3-week period of repeated subculturing at a 1:3 ratio, three times per week, in 10-cm culture dishes. For clonal analysis, the selected cells were plated in 96-well plates at one cell per 100 μL by limiting dilution in DMEM-LG supplemented with 10% FBS and P/S. Briefly, only wells containing one cell per well were selected by visual inspection 24 hrs after plating, and were cultured for 14 days. Among 16 clones derived from a single cell, two clones (CD34+ A34P1 and CD34- A34N3) were finally selected based on microscopic examination of cell morphology, proliferation, and expression levels of GFP and hTERT.

### Phenotypic characterization of primary and hTERT-immortalized mADSCs

For immunohistochemistry, primary and hTERT-immortalized mADSCs plated onto coverslips coated with 0.1% gelatin (Sigma Aldrich, St. Louis, MO, USA) in a 24-well plate were fixed with 4% paraformaldehyde in PBS for 10 mins and washed with PBS + 0.1% Tween-20 (PBST). The cells were blocked in 5% normal goat serum (NGS; Invitrogen) in PBST for 30 mins, and stained for 30 mins with the following primary antibodies: CD14, CD31, CD34, CD44, CD45, CD71, CD106, and Sca-1 (all from BD Biosciences). The cells were stained with Alexa Fluor 594-conjugated goat anti-rat antibodies (1:1,000, Molecular Probes, Eugene, OR, USA) for 30 mins and washed three times in PBST. The nuclei were stained with 4′,6-diamidino-2-phenylindole dihydrochloride (DAPI; Sigma-Aldrich) and the cells were mounted using fluorescent mounting medium (DAKO, Glostrup, Denmark). Fluorescence images were obtained using the TEFM Epi-Fluorescence system attached to an inverted microscope (Olympus, Tokyo, Japan). For flow cytometry, primary and hTERT-immortalized mADSCs were fixed with 4% paraformaldehyde in PBS for 10 mins at room temperature (RT). The cells were subsequently incubated for 20 mins at 4°C with the following primary antibodies: CD14, CD31, CD34, CD44, CD45, CD71, CD106, and Sca-1 (all from BD Biosciences). After washing twice with PBS + 2% FBS, the cells were incubated with fluorescein isothiocyanate (FITC)-conjugated goat anti-rat antibodies (Sigma Aldrich) for 15 mins at 4°C. For the control experiments, the cells were stained with secondary antibodies only. After washing twice with PBS + 2% FBS, thirty thousand cells for each sample were analyzed on a FACS Calibur flow cytometer (BD Biosciences) and by Cell Quest Pro software (BD Biosciences).

### Differentiation potential of hTERT-immortalized mADSCs

Adipogenic differentiation of hTERT-immortalized mADSCs was induced by incubation in DMEM-LG supplemented with 5% FBS and P/S, 1 μM dexamethasone, 100 μM indomethacin, 0.5 μM methyl-isobutylxanthin (all from Sigma), and 10 μg/mL insulin for 10 days. After 10 days of culture, Oil Red O (Sigma Aldrich) staining was employed to assess the degree of lipid accumulation within the cells. To quantify Oil Red O-stained lipid droplets, the cells were incubated for 10 mins with shaking after adding 100% isopropanol. The reaction product was quantified by measuring the absorbance at 500 nm using an ELISA reader (Molecular Devices, PA, USA). Data were analyzed using SoftMax Pro quantification of absorbance analysis software (Molecular Devices). Osteogenic differentiation of hTERT-immortalized mADSCs was induced by incubation in DMEM-LG supplemented with 10% FBS and P/S, 1 μM dexamethasone, 10 mM glycerophosphate, and 50 μM ascorbic acid (all from Sigma Aldrich) for 21 days. Osteogenic differentiation was determined by staining with Alizarin Red S (Sigma Aldrich). For quantification of Alizarin Red S staining, the cells were incubated for 10 mins with shaking after adding dimethyl sulfoxide. The reaction product was quantified by measuring the absorbance at 405 nm using an ELISA reader (Molecular Devices). Data were analyzed using SoftMax Pro quantification of absorbance analysis software (Molecular Devices). For cardiomyogenic differentiation, 2 × 10^4^ cells of hTERT-immortalized mADSCs were plated into 12-well plates containing 0.1% gelatin-coated glass coverslips and cultured in cardiomyogenic differentiation medium (DMEM-LG, 10% FBS, P/S, 1 mg/mL insulin, 0.55 mg/mL transferrin, 0.5 μg/mL sodium selenite, 50 mg/mL bovine serum albumin, 0.47 μg/mL linoleic acid, 10^−4^ M ascorbate, 10^−9^ M dexamethasone) with 10 μM 5-azacytidine (all from Sigma Aldrich) for 3 days. The cells were induced by incubation in cardiomyogenic differentiation medium for 3 weeks. The cells were exposed to 100 ng/mL Trichostatin A (TSA; Sigma Aldrich) for 24 hrs on day 14 [21]. Endothelial differentiation of hTERT-immortalized mADSCs was induced by incubation in 60% DMEM-LG and 40% MCDB-201, supplemented with 1 × insulin-transferrin-selenium, 1 × linoleic acid-BSA, 10^−8^ M dexamethasone, 10^−4^ M ascorbic acid 2-phosphate (all from Sigma Aldrich), P/S plus 20 ng/mL vascular endothelial growth factor (VEGF; R&D Systems, Minneapolis, MN, USA) for 3 weeks. To assess cardiomyogenic or endothelial differentiation, the cells were incubated overnight at 4°C with the following primary antibodies; anti-cardiac troponin T (cTnT; RV-C2-s, DSHB, Iowa City, IA, USA), anti α-actinin antibody (Santa Cruz Biotechnology, Santa Cruz, CA, USA) and anti-von Willebrand factor (vWF; DAKO). Then, the cells were stained with Alexa Fluor 488- or 594-conjugated secondary antibodies (Molecular Probes) for 1 hr at RT. The nuclei were stained with DAPI (Sigma Aldrich). The cells were mounted with fluorescent mounting medium (DAKO). The fluorescence images were obtained with the TE-FM Epi-Fluorescence system attached to an inverted microscope (Olympus).

### Real-time PCR

Total RNAs were extracted from hTERT-immortalized mADSCs using Trizol reagent (Invitrogen). The concentration of total RNAs was determined using a Nanodrop 1000 spectrophotometer (Thermo Scientific, Waltham, MA, USA). cDNA synthesis and real-time PCR was performed as previously described [22]. The primers used for real-time PCR were as follows: 5'- GGCTCACTTCGAGAACAGGA -3', 5'- TCATTGCGAATACGCTGCT-3' (mcTnT, 108 bp); 5'-CCTGCGGCCTCTACATGA-3', 5'- AGGGTCTCACCAGCAGGA-3' (mGATA4, 136 bp); 5'-CGGAAGAGTGTCTGGAGCAA-3', 5'-GGATGAAGCGGAGTCTGGA-3' (hTERT, 145 bp); 5'-TTCACCACCATGGAGAAGGC-3', 5'-GGCATGGACTGTGGTCATGA-3' (mGAPDH, 236 bp). Real-time PCR data were pooled from three independent experiments. Relative gene expression levels were quantified based on Ct and normalized to the reference gene, GAPDH.

### WST-1 proliferation assay

hTERT-immortalized mADSCs were plated at a density of 1 × 10^3^ cells/well in a 96-well plate, cultured in Mesencult MSC Basal Medium supplemented with 10% Mesencult MSC Stimulatory Supplements (StemCell Technologies) and P/S, and were analyzed at days 5, 7, and 9 by the WST-1 assay (Roche Applied Science, Mannheim, Germany). In brief, the cells were incubated at a concentration of 10 μM of WST-1 for 2 hrs. The cells were then incubated until color development was sufficient for photometric detection. The reaction product was quantified by measuring absorbance at 440 and 690 nm using an ELISA reader (Molecular Devices). Data were analyzed using SoftMax Pro quantification of absorbance analysis software (Molecular Devices).

### Comparative analysis of paracrine factors in primary and hTERT-immortalized mADSCs

To prepare conditioned medium (CM), primary and hTERT-immortalized mADSCs were seeded at 1 × 10^5^ cells in 100-mm tissue culture dishes and allowed to reach ~80% confluence in Mesencult Complete Medium. The medium was then changed to Mesencult MSC Basal Medium supplemented with 2% FBS and P/S, and further cultured for 24 hrs. Next, CM was collected, filtered through a 0.22 μm pore size filter (Merck Millipore, Billerica, MA, USA), and stored at 4°C until use. A mouse cytokine antibody array (RayBiotech, Norcross, GA, USA) that included 21 cytokines in duplicate and appropriate positive and negative controls was customized. The analyzed proteins included epidermal growth factor (EGF), TGF-β1, hepatocyte growth factor (HGF), insulin-like growth factor 1 (IGF-1), IGF-2, monocyte chemoattractant protein-1 (MCP-1), VEGF, stromal cell-derived factor 1 (SDF-1), basic fibroblast growth factor (bFGF), E-Cadherin, HGF receptor (HGFR), IFN-γ, IL-10, tumor necrosis factor-alpha (TNF-ɑ), IL-6, IL-1ɑ, IL-1β, IL-1Ra, leptin, cardiotrophin-1 (CT-1), and MCP-5. Densitometric quantification of blotting spots was performed using Quantity One software (Bio-Rad, Hercules, CA, USA).

### Costimulation assay

Female Sprague-Dawley (SD) rats weighing 180–200 g were purchased from Orient Experimental Animal Laboratory (Gyeonggi, Korea). The SD rats were given an IP injection anesthetized with a mixture of ketamine (60 mg/kg) and xylazine hydrochloride (7.5 mg/kg). Splenocytes were isolated by filtration through a 100 μm nylon mesh from female SD rat spleens. Erythrocytes were removed using 160 mM NH_4_Cl for 10 mins, followed by two washes in RPMI 1640 (Lonza, Walkersville, MD, USA) supplemented with 10% FBS and P/S. hTERT-immortalized mADSCs (5 × 10^4^/well) were seeded to confluency in 96-well plates 24 hrs before coculturing with rat splenocytes at 1:2 and 1:4 ratios. Cocultures were kept for 24 hrs in the presence of Phytohemagglutinin (PHA; 10 μg/mL, Sigma Aldrich) and anti-rat CD28 (rCD28; 5 μg/mL, BD Biosciences). Rat splenocytes treated with PHA and rCD28 were included as a positive control. After 24 hrs of coculture, supernatants were collected and assayed for rIL-2 by ELISA (R&D Systems). Each assay was done in triplicates. The reaction product was quantified by measuring absorbance at 450 and 540 nm using an ELISA reader (Molecular Devices).

### AMI model and cell transplantation

Female SD rats weighing 180–200 g were given an IP injection anesthetized with a mixture of ketamine (60 mg/kg) and xylazine hydrochloride (7.5 mg/kg). An 18-gauge angiocatheter (BD Bioscience) was utilized as an intubation tube throughout the procedure. The left coronary artery of the heart was ligated with a 6–0 silk suture, 5-mm from the left coronary atrial appendage. After confirmation of the presence of MI, 1 × 10^6^ hTERT-immortalized mADSCs prepared in 100 μL of culture medium was injected at three peri-infarct areas. 100 μL of culture medium was injected as a control treatment. After cell transplantation, the chest wall, muscle layers, and skin were closed with 3–0 silk sutures. All AMI induced rats were continuously monitored from surgery to recovery. For sacrifice, rats were euthanized by IP injection with the mixture of ketamine (60 mg/kg) and xylazine hydrochloride (7.5 mg/kg).

### Echocardiographic analysis

Echocardiography was performed at 1, 7, 14, and 28 days after cell transplantation with a commercially available Vivid 7 echocardiography system (GE Healthcare, Wauwatosa, WI, USA) with a 10 MHz small linear array transducer for animal research was used. The depth of the window was adjusted to 2 cm, and parasternal long- and short-axis views were obtained. SD rats were anesthetized with a mixture of ketamine (60 mg/kg) and xylazine hydrochloride (7.5 mg/kg). Posterior wall thickness in diastole (PWTd), posterior wall thickness in systole (PWTs), left ventricular end of diastolic volume (LVEDV), left ventricular end-systolic volume (LVESV), and left ventricular anterior wall thickening at the end-diastole data were obtained by a two dimensional targeted M-mode view. LV volume and ejection fraction (EF) was calculated using the modified Simpson’s method from parasternal views. All measurements were taken over 2 consecutive cardiac cycles and averaged. All measurements were performed by an experienced cardiologist who was blind to the study group. Each value was the average of two measurements. The percentage of fractional shortening (FS) was computed as representative of systolic function; EF was calculated as EF (%) = [end diastolic volumes (Voldia)—end-systolic volumes (Volsys)] / Voldia. Voldia and Volsys were calculated by manually drawing endocardial contours at end-diastolic and end-systolic phases in the apical two chambers view using the modified Simpson’s rule.

### Detection of circulating IL-6 and MCP-1 levels

After echocardiographic analysis, rat blood samples were collected from the tail vein into EDTA-containing tubes using a 24-gauge catheter (BD Bioscience) at 1, 7, 14, and 28 days after cell transplantation. Circulating IL-6, and MCP-1 levels were measured using microsphere-based multiplex assays (MILLIPLEX MAP Kit, Rat Cytokine/Chemokine Magnetic Bead Panel; EMD Millipore, Darmstadt, Germany) according to the manufacturer's instructions.

### Immunohistochemical analysis of tissue sections

Masson's trichrome staining was performed using the Trichrome Stain kit (Sigma Aldrich) with the following modifications: nuclei were stained with Celestine Blue solution followed by Gill’s Hematoxylin staining, and tissue was incubated for 1 hr in Bouin’s solution before muscle staining with Biebrich Scarlet-Acid Fuchsin (Sigma Aldrich). For fluorescence in situ hybridization (FISH), prepared tissue slides were dehydrated and incubated with probe specific to whole mouse Y chromosome (Empire genomics, Buffalo, NY, USA) for 10 hrs at 37°C in a humidified chamber. Tissues were then incubated with DAPI.

At 72 hrs after cell transplantation, the numbers of infiltrating CD8+ T cells and CD68+ macrophages were assessed by incubating sections (5 or 7 μm thickness) with CD8 (Biolegend, San Diego, CA, USA), CD68 (Abcam, Cambridge, England) and GFP+ antibody (Abcam). Slides were washed and then incubated with Alexa Fluor 488- or 594-conjugated secondary antibodies (all from Molecular Probes) for 30 mins. Finally, sections were incubated with DAPI for 1 min. Quantification of GFP+/CD8+ or GFP+/CD68+ cells was achieved using Image-Pro 7.0 software (Mediacybernetics, Rockville, MD, USA) on 8 sections (two fields per section, two sections per heart, *n* = 2 for each group) in the border zones around the infarct site.

At day 28 after cell transplantation, angiogenesis was assessed by incubating sections (5 or 7 μm thickness) overnight with GFP antibody (Abcam) and with anti-vWF (DAKO). Slides were washed and then incubated with Alexa Fluor 488- or 594-conjugated secondary antibodies (all from Molecular Probes) for 30 mins. Finally, sections were incubated with DAPI for 1 min. Fluorescence images of sections were photographed at a magnification of 400x with the TE-FM Epi-Fluorescence system attached to an inverted microscope (Olympus). Quantification of GFP+/vWF+ cells was achieved using Image-Pro software on 20 sections (two fields per section, two sections per heart, *n* = 5 for each group) in the border zones around the infarct site.

### Statistical analysis

All statistical values are expressed as the mean ± standard deviation (SD). Significant differences between the means were determined using the Student’s t-test or by analysis of variance followed by the Student–Newman–Keuls test. Statistical significance was set at *p* < 0.05. All statistical analyses were performed using SigmaStat 3.5 software (SPSS, Chicago, IL, USA).

## Results

### Establishment of hTERT-immortalized mADSC lines

**Fig 1:**
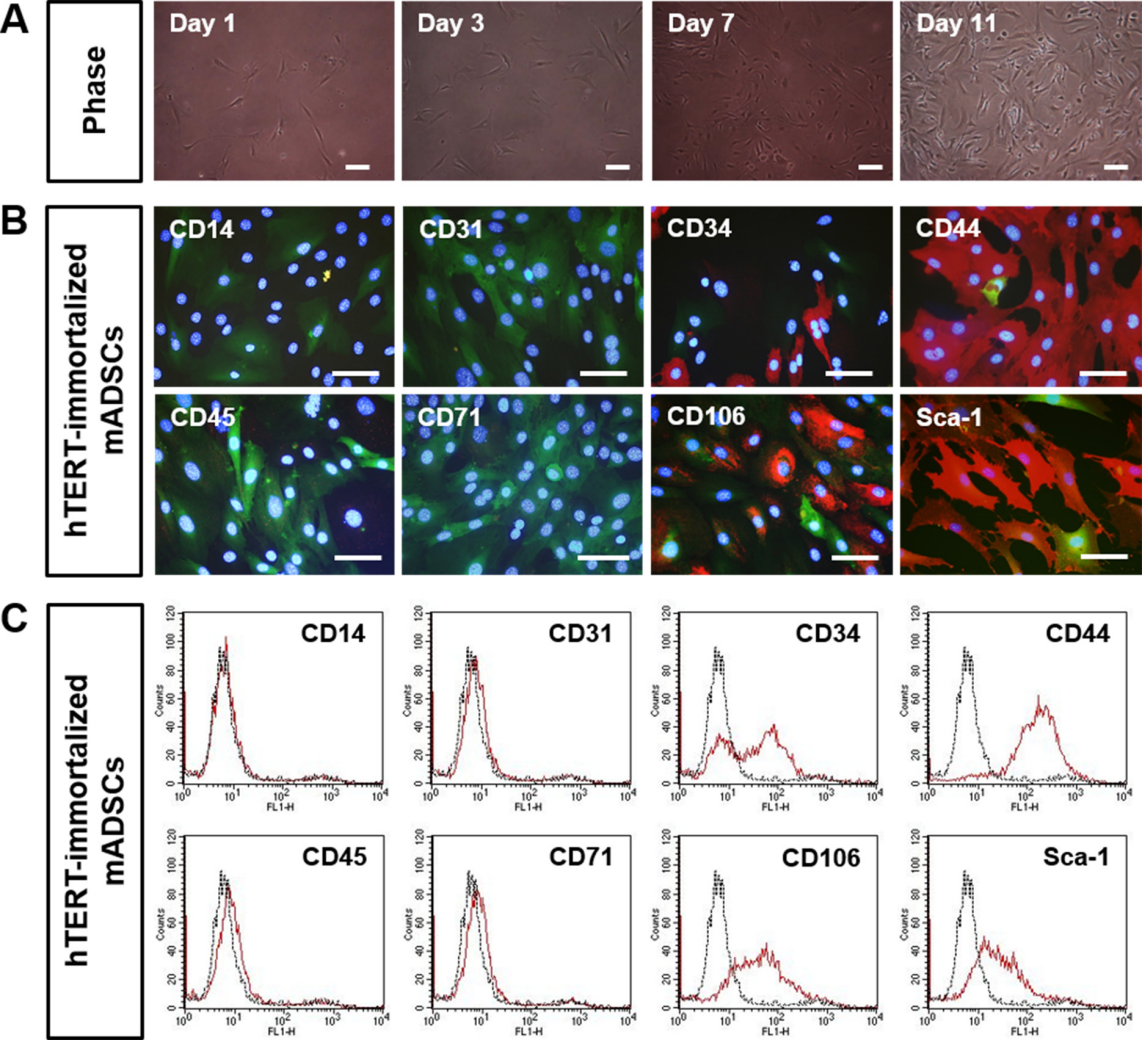
Phenotypic characterization of hTERT-immortalized mADSCs. Primary mADSCs were infected with a retroviral vector harboring hTERT-IRES-EGFP, and selected for about 3 months with puromycin. (A) Morphology of hTERT-immortalized mADSCs. Morphological properties of hTERT-immortalized mADSCs were analyzed at 1, 3, 7, and 11 days after initiation of cultures. (B) hTERT-immortalized mADSCs were stained with primary antibodies against CD14, CD31, CD34, CD44, CD45, CD71, CD106, and Sca-1, and were visualized with Alexa Fluor-594 conjugated anti-rat antibodies (red). Nuclei were stained with DAPI. Immunofluorescence staining of hTERT-immortalized mADSCs shows positive expression for CD34, CD44, CD106, and Sca-1. Scale bars = 100 μm. (C) Flow cytometric analysis of hTERT-immortalized mADSCs shows positive expression for CD34, CD44, CD106, and Sca-1. Scale bars in A and B = 100 μm.

Primary mADSCs isolated from inguinal adipose tissue showed fibroblast-like morphology (S1A Fig). For the phenotypic characterization, primary mADSCs were stained with antibodies to stem cell or cell lineage markers by immunohistochemistry and flow cytometry. Primary mADSCs were positive for CD34, CD44, CD106, and Sca-1, whereas they were negative for CD14, CD31, CD45, and CD71 (S1A and S1B Fig). In order to immortalize mADSCs with the hTERT gene, freshly isolated primary mADSCs were infected with retroviruses harboring hTERT-IRES-EGFP. At 72 hrs after infection with retroviruses, GFP-positive mADSCs were detected by fluorescence microscopy. After selecting mADSCs in a medium containing puromycin for about 3 months, the cell phenotypic characteristics were investigated. hTERT-immortalized mADSCs showed fibroblast-like shapes morphologically similar to the primary mADSCs (Fig 1A). hTERT-immortalized mADSCs were positive for CD34, CD44, CD106, and Sca-1 (Fig 1B and 1C), showing that they were also phenotypically similar to the primary mADSCs. Interestingly, we found that approximately half of hTERT-immortalized mADSCs was positive for CD34, a hematopoietic stem cell marker (Fig 1B and 1C). Therefore, we further selected at a single cell level by limiting dilution in 96-well cell culture plates in order to isolate CD34+ and CD34- mADSC lines (S1 Table).

### Evaluation of stem cell potency of hTERT-immortalized CD34+ and CD34- mADSC lines

**Fig 2:**
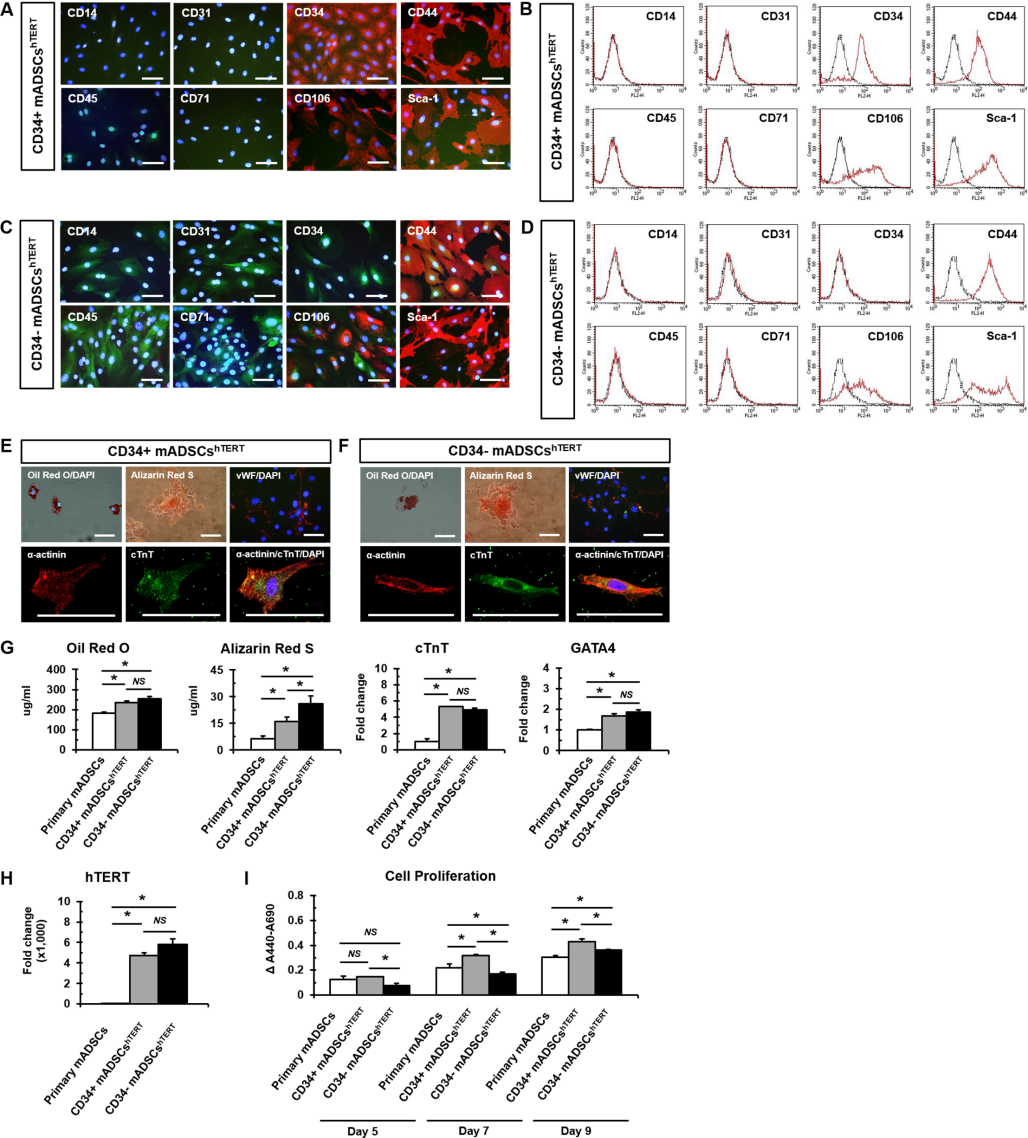
hTERT-immortalized mADSCs exhibit multi-lineage differentiation capacity. Immunofluorescence staining (A) and flow cytometric analysis (B) of CD34+ mADSCs^hTERT^ show positive expression of CD34, CD44, CD106, and Sca-1. Immunofluorescence staining (C) and flow cytometric analysis (D) of CD34- mADSCs^hTERT^ show positive expression for CD44, CD106, and Sca-1. CD34+ (E) and CD34- (F) mADSCs^hTERT^ exhibit adipogenic (Oil Red O positive), osteogenic (Alizarin Red S positive), endothelial (vWF positive), and cardiomyogenic differentiation potentials (α-actinin positive and cTnT positive). Nuclei were stained with DAPI (blue). (G) Adipogenic differentiation of primary mADSCs, CD34+, and CD34- mADSCs^hTERT^ was visualized by Oil Red O staining and was quantified by measuring the absorbance at 500 nm in a microplate reader. Osteogenic differentiation of primary mADSCs, CD34+, and CD34- mADSCs^hTERT^ was visualized by Alizarin Red S staining and was quantified by measuring the absorbance at 405 nm in a microplate reader. Cardiomyogenic differentiation of primary mADSCs, CD34+, and CD34- mADSCs^hTERT^ was analyzed by quantitative real-time PCR with primers for cardiomyocyte-specific markers (cTnT and GATA4). Data shown represent mean ± SD from three independent experiments (*p < 0.05; *NS*, not significant). (H) Real-time PCR was performed to detect transduced hTERT transcripts in CD34+ and CD34- mADSCs^hTERT^. Primary mADSCs were used as a negative control. Data shown represent mean ± SD from three independent experiments (*p < 0.05; *NS*, not significant). (I) Cell proliferation of primary mADSCs, CD34+, and CD34- mADSCs^hTERT^ was evaluated by WST-1 assay at 5, 7, and 11 days after seeding. Data shown represent mean ± SD from three independent experiments (*p < 0.05; *NS*, not significant). Scale bars in A, C, E, and F = 100 μm.

For further studies, clone A34P1, designated as CD34+ mADSCs^hTERT^, was finally selected out of seven CD34+ mADSC lines, and clone A34N3, designated as CD34- mADSCs^hTERT^, was also selected from nine CD34- mADSC lines based on the morphology, proliferation rate, and GFP expression of the cells (S1 Table). Phenotypic characterization of the selected CD34+ and CD34- mADSCs^hTERT^ was further evaluated by immunostaining and flow cytometry with different cell surface antibodies. CD34+ and CD34- mADSCs^hTERT^ were positive for CD44, CD106, and Sca-1 (Fig 2A–2D), and they were negative for CD14, CD31, CD45, and CD71 (Fig 2A–2D), indicating that they represent phenotypic characteristics similar to that of primary mADSCs.

Multi-potency of CD34+ and CD34- mADSCs^hTERT^ was investigated by inducing mADSC into adipogenic, osteogenic, endothelial, and cardiomyogenic lineages, which were confirmed by Oil Red O staining, Alizarin Red S staining, vWF expression, and α-actinin/cTnT expression, respectively. CD34+ and CD34- mADSCs^hTERT^ demonstrated differentiation potential for four different cell types (Fig 2E and 2F). Fig 2G shows that CD34+ and CD34- mADSCs^hTERT^ exhibited significantly higher adipogenic, osteogenic, endothelial, and cardiomyogenic differentiation potentials compared to those of primary mADSCs (passage 3). Interestingly, CD34- mADSCs^hTERT^ exhibited a significantly higher osteogenic differentiation potential compared to CD34+ mADSCs^hTERT^ (Fig 2G). As expected, transduced hTERT transcripts were strongly expressed in both CD34+ and CD34- mADSCs^hTERT^ (Fig 2H), indicating that the stemness of CD34+ and CD34- mADSCs^hTERT^ was sustained by ectopic hTERT activity.

CD34+ and CD34- mADSCs^hTERT^ were cultured for about 4 months, which corresponded to around 100 population doublings (PDs) before performing the WST-1 assay. Primary mADSCs (passage 3) and CD34+ mADSCs^hTERT^ showed significantly higher proliferation rates compared to CD34- mADSCs^hTERT^ at 5 days after seeding (Fig 2I). CD34+ mADSCs^hTERT^ also demonstrated a significantly higher proliferation rate compared to primary mADSCs and CD34- mADSCs^hTERT^ at 7 and 9 days after seeding (Fig 2I). Primary mADSCs showed a significantly higher proliferation rate compared to CD34- mADSCs^hTERT^ at 7 days after seeding, whereas their proliferation rate became significantly lower than that of CD34+ mADSCs^hTERT^ at 9 days after seeding (Fig 2I). These results demonstrated that CD34+ and CD34- mADSCs^hTERT^ sustained a higher proliferation ability compared to primary mADSCs, even after long-term culture exceeding 100 PDs.

### Comparison of paracrine factors secreted from primary mADSCs and CD34+ and CD34- mADSCs^hTERT^

**Fig 3:**
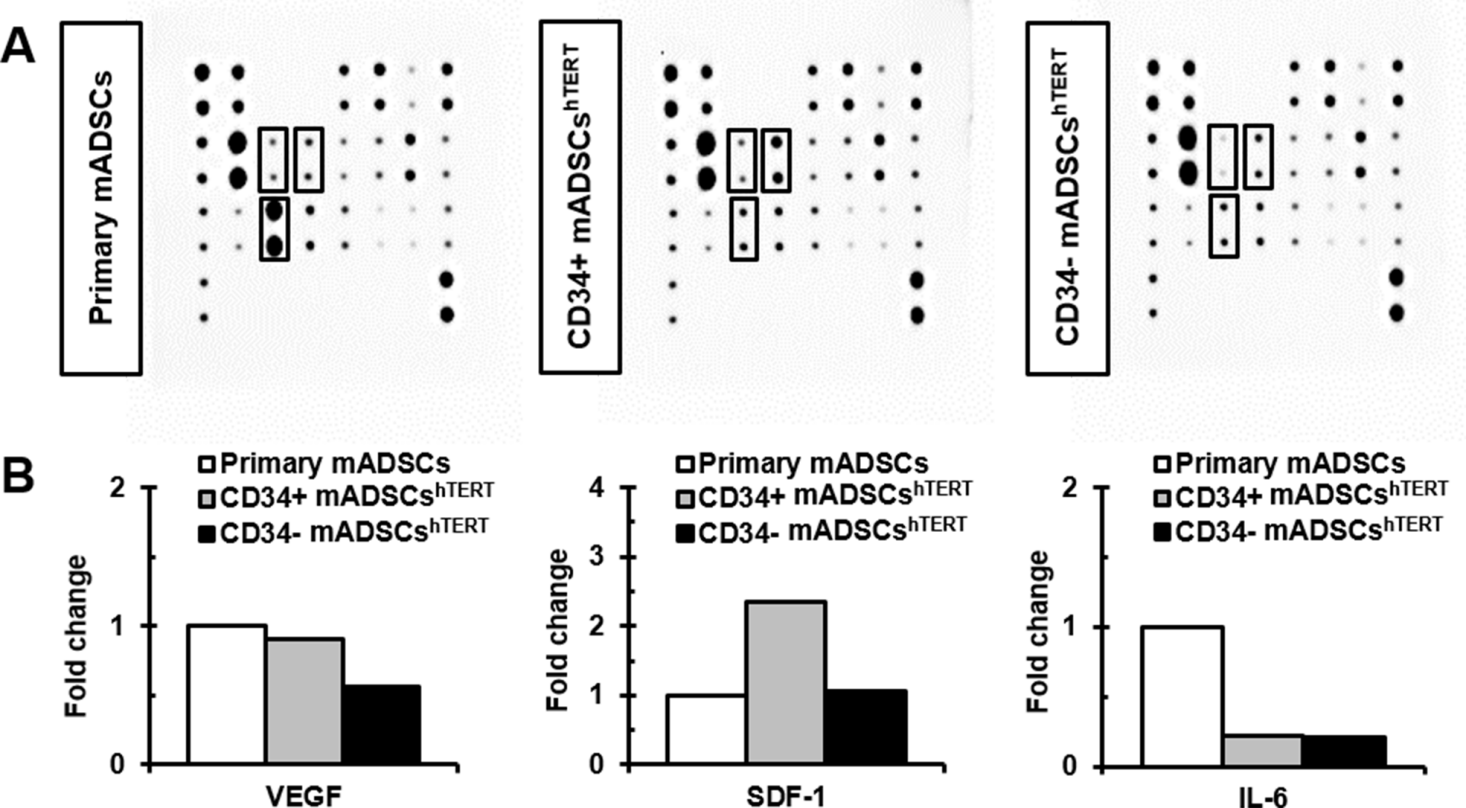
Determination of paracrine factors secreted from primary mADSCs and CD34+ and CD34- mADSCs^hTERT^. (A) CM secreted from primary mADSCs, CD34+, and CD34- mADSCs^hTERT^ was subjected to a mouse cytokine antibody array detecting 21 cytokines in duplicate. Solid lined boxes indicate differentially secreted paracrine factors among primary mADSCs, CD34+, and CD34- mADSCs^hTERT^. (B) Differentially expressed paracrine factors among primary mADSCs, CD34+, and CD34- mADSCs^hTERT^ were subjected to densitometry and are presented as fold changes for individual cytokines, taking primary mADSCs as a one-fold value.

A mouse cytokine antibody array (S2 Fig) containing 21 growth factors and inflammatory cytokines was used to identify which paracrine factors were secreted from primary mADSCs, CD34+, and CD34- mADSCs^hTERT^. The dominant growth factors and cytokines secreted from primary mADSCs, CD34+, and CD34- mADSCs^hTERT^ were EGF, TGF-β1, IGF-1, IGF-2, MCP-1, and HGFR (Fig 3A). Among these proteins, MCP-1 was the predominant factor secreted from primary mADSCs, CD34+, and CD34- mADSCs^hTERT^ (Fig 3A). Inflammatory cytokines such as IL-6, IL-10, IL-1ɑ, IL-1β, and MCP-5 were abundant in primary mADSCs CM, CD34+ mADSCs^hTERT^ CM, and CD34- mADSCs^hTERT^ CM (Fig 3A). We found that VEGF, SDF-1, and IL-6 were differentially expressed among primary mADSCs, CD34+, and CD34- mADSCs^hTERT^ (Fig 3A). VEGF levels were reduced by 1.6-fold in CD34- mADSCs^hTERT^ CM compared to primary mADSCs CM and CD34+ mADSCs^hTERT^ CM (Fig 3B). In contrast, SDF-1 levels were increased by approximately 2.3-fold in CD34+ mADSCs^hTERT^ CM compared to primary mADSCs CM and CD34- mADSCs^hTERT^ CM (Fig 3B). Interestingly, secretion levels of IL-6, a pro-inflammatory cytokine, were severely reduced in both CD34+ mADSCs^hTERT^ CM (4.5-fold) and CD34- mADSCs^hTERT^ CM (4.8-fold) compared to that of primary mADSCs CM (Fig 3B).

### Transplantation of CD34+ and CD34- mADSCs^hTERT^ into infarcted myocardium increased cardiac function and reduced infarct size

**Fig 4:**
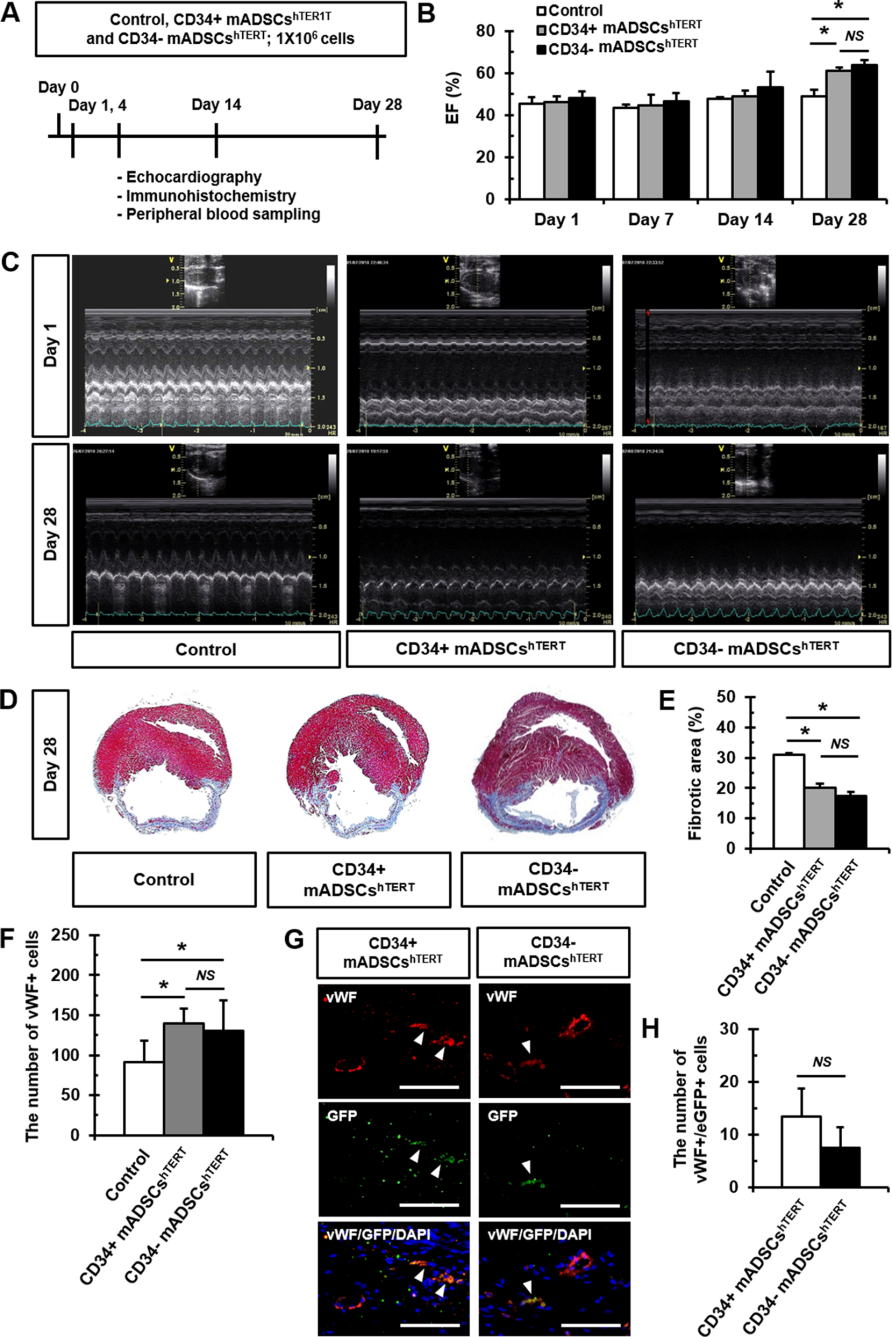
Transplantation of CD34+ and CD34- mADSCs^hTERT^ into a rat AMI model improved cardiac function and reduced fibrosis partly via endothelial differentiation. (A) Schematic diagram of the study protocol. 1 × 10^6^ CD34+ and CD34- mADSCs^hTERT^ were transplanted into the peri-infarct region of a rat AMI model. Echocardiography was performed at 1, 7, 14, and 28 days after cell transplantation. (B) LVEF was significantly improved in CD34+ and CD34- mADSCs^hTERT^ groups at day 28 after transplantation. Data shown represent mean ± SD (*n* = 10, *p < 0.05; *NS*, not significant). (C) Representative images of 2D echocardiography demonstrating improved LVEF in CD34+ and CD34- mADSCs^hTERT^ groups at day 28 after transplantation. (D) Representative Masson’s trichrome-stained images showing reduced fibrotic areas in CD34+ and CD34- mADSCs^hTERT^ groups at day 28 after transplantation. (E) Myocardial fibrosis was quantified at day 28 after cell transplantation. Data shown represent mean ± SD (*n* = 10, *p < 0.05, **p < 0.01; *NS*, not significant). (F) The number of vWF+ cells in the infarcted hearts of CD34+ and CD34- mADSCs^hTERT^ injected rats was quantified using Image-Pro 7.0 software at day 28 after cell transplantation. Data shown represent mean ± SD (*NS*, not significant) on 20 sections. (G) Representative images showing GFP+ (green)/vWF+ (red) cells (arrowheads) in blood vessels of the infarcted hearts of CD34+ and CD34- mADSCs^hTERT^ injected rats at day 28 after cell transplantation. (H) The number of GFP+/vWF+ cells in the infarcted hearts of CD34+ and CD34- mADSCs^hTERT^ injected rats was quantified using Image-Pro 7.0 software at day 28 after cell transplantation. Data shown represent mean ± SD (*NS*, not significant) on 20 sections (two fields per section, two sections per heart, *n* = 5 for each group). Scale bars = 100 μm.

CD34+ and CD34- mADSCs^hTERT^ were transplanted into the peri-infarct region of a rat AMI model to investigate their roles in cardiac regeneration. AMI-induced rats injected with an equivalent volume of medium with no cells were used as a control. Cardiac function was evaluated by echocardiography at 1, 7, 14, and 28 days following cell transplantation (Fig 4A). CD34+ and CD34- mADSCs^hTERT^ showed significant increases in left ventricular ejection fraction (LVEF) at 28 days compared to 1 day (Fig 4B and 4C, and S2 Table). No significant differences in body weight changes were found between groups from day 0 to day 28 (S3A Fig). Delta LVEFs defined as changes in LVEF from baseline to post-cell transplantation were significantly improved in CD34+ and CD34- mADSCs^hTERT^ groups compared to the medium-injected control group at day 28 after cell transplantation (S3B Fig). However, no significant differences in LVEF between CD34+ and CD34- mADSCs^hTERT^ groups were found. In addition, significant increases in PWTd and PWTs were observed at 28 days in CD34+ and CD34- mADSCs^hTERT^ groups compared to the medium-injected control group (S2 Table).

To investigate the effect of CD34+ and CD34- mADSCs^hTERT^ transplantation on the degree of fibrosis, we carried out Masson's trichrome staining on tissue sections after transplantation. Masson's trichrome staining showed significant reduction in fibrosis after AMI in CD34+ mADSCs^hTERT^ (20.13±3.25%) and CD34- mADSCs^hTERT^ (17.46±3.00%) compared to the medium-injected control group (30.86±1.62%) (Fig 4E).

### CD34+ and CD34- mADSCs^hTERT^ transplanted into infarcted myocardium were differentiated into endothelial cells

To determine whether transplanted cells could differentiate into endothelial cells or cardiomyocytes in the infarct heart, tissue sections were double-stained for GFP and cardiac- or endothelial-specific markers. Engraftment of CD34+ and CD34- mADSCs^hTERT^ (male mouse origin) was confirmed in the recipient female rats by fluorescence staining for Y chromosome in the infarcted heart at day 28 after cell transplantation (S3C Fig). The number of vWF+ cells was significantly increased in both CD34+ and CD34- mADSCs^hTERT^ transplanted groups compared to the medium-injected control group at 28 days after cell transplantation (Fig 4F). Furthermore, GFP+/vWF+ cells were also found in both CD34+ and CD34- mADSCs^hTERT^ groups at 28 days after cell transplantation, demonstrating that transplanted CD34+ and CD34- mADSCs^hTERT^ were differentiated into endothelial cells in the infarcted myocardium (Fig 4G). However, there were no significant differences between CD34+ (13.38±5.37) and CD34- mADSCs^hTERT^-transplanted groups (7.50±3.83) (Fig 4H). However, no GFP+/cTnT+ cells were found in either CD34+ or CD34- mADSCs^hTERT^ groups at 28 days after cell transplantation. We found that transplantation of primary mADSCs, CD34+, and CD34- mADSCs^hTERT^ into nude mice did not form tumors even up to 6 months after transplantation (S4A Fig). In addition, significant differences in body weight changes after transplantation of primary mADSCs, CD34+, and CD34- mADSCs^hTERT^ into nude mice were not found between groups (S4B Fig).

### Transplantation of CD34+ and CD34- mADSCs^hTERT^ into a rat AMI model reduced circulating pro-inflammatory cytokine levels

**Fig 5:**
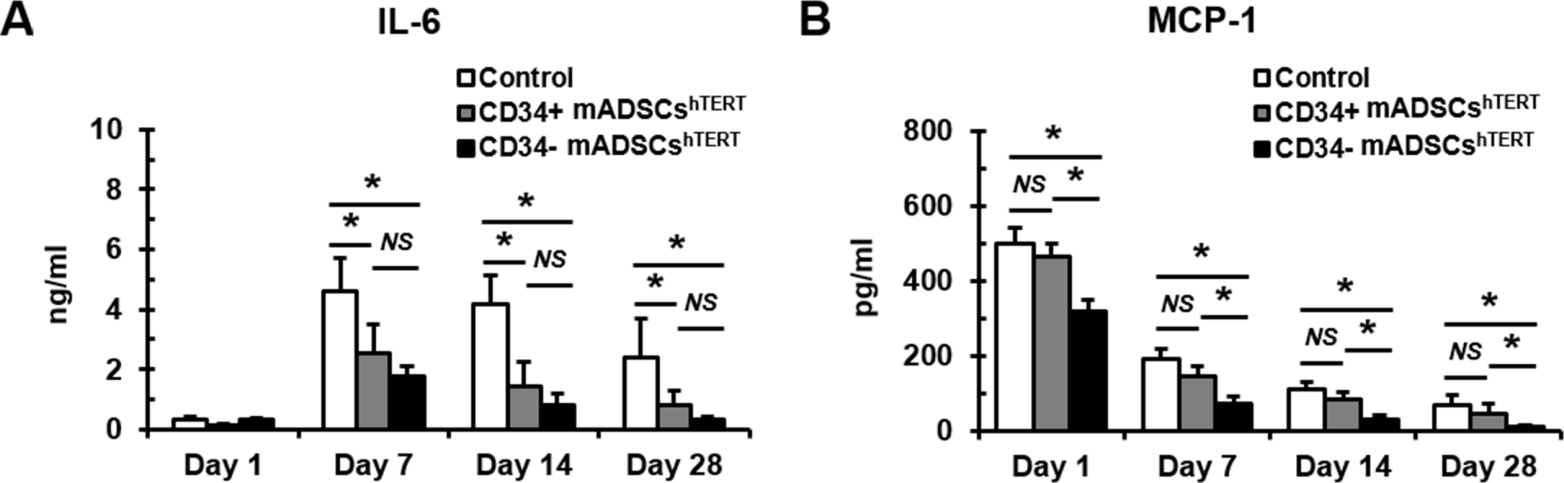
Transplantation of CD34+ and CD34- mADSCs^hTERT^ into a rat AMI model significantly reduced circulating pro-inflammatory cytokine levels. Circulating IL-6 (A) and MCP-1 (B) levels were measured using Milliplex Max kit from rat plasma samples obtained from tail vein at days 1, 7, 14, and 28 after cell transplantation. Data shown represent mean ± SD (*n* = 10, *p < 0.05; *NS*, not significant).

To further examine whether transplantation of CD34+ and CD34- mADSCs^hTERT^ into AMI rats alters the systemic inflammatory response, we analyzed changes in circulating levels of inflammatory cytokines (IL-6 and MCP-1) at 1, 7, 14, and 28 days following cell transplantation. Significant decreases in circulating IL-6 levels were found at 7, 14, and 28 days after transplantation of CD34+ and CD34- mADSCs^hTERT^ groups compared to the medium-injected control group (Fig 5A). Transplantation of CD34- mADSCs^hTERT^ significantly reduced circulating MCP-1 levels at 1, 7, 14, and 28 days compared to the medium-injected control group and CD34+ mADSCs^hTERT^ group (Fig 5B).

To determine the rat host responses to xenotransplanted CD34+ and CD34- mADSCs^hTERT^, we assessed whether CD34+ or CD34- mADSCs^hTERT^ could provide costimulatory signals to rat splenocytes through the CD28 pathway [23–25]. CD34+ or CD34- mADSCs^hTERT^ with rat splenocytes were cocultured in the presence of PHA and rCD28 determined rIL-2 secretion in culture supernatants. We measured rIL-2 release after 24 hrs of coculture at 1:2 and 1:4 ratios (S5A Fig). Coculture of CD34+ mADSCs^hTERT^ to rat splenocytes activated by PHA and rCD28 did not significantly affect rIL-2 secretion at both 1:2 and 1:4 ratios (S5A Fig) compared to the control group. On the contrary, coculture of CD34- mADSCs^hTERT^ to rat splenocytes activated by PHA and rCD28 induced moderately but significantly rIL-2 secretion at both 1:2 and 1:4 ratios (S5A Fig) compared to the control and CD34+ mADSCs^hTERT^ groups. Furthermore, we also investigated the numbers of infiltrating CD8+ T cells and CD68+ macrophages at 72 hrs after xenotransplantation of CD34+ or CD34- mADSCs^hTERT^ into the infarcted rat hearts. There were no significant differences between the numbers of CD8+ T cells and CD68+ macrophages in the medium-injected control, CD34+ and CD34- mADSCs^hTERT^ transplanted groups (S5B Fig). Severe migration of CD8+ T cells and CD68+ macrophages to the transplanted GFP+ cells was also not observed (S5C Fig). Taken together, these results indicate that CD34+ or CD34- mADSCs^hTERT^ have immunomodulatory effects although they provoked different immune responses in a cell type-specific manner.

## Discussion

Counter et al. [26] reported that alteration of the carboxyl terminus of TERT does not affect telomerase activity but impedes the ability of this enzyme to maintain telomeres, resulting in the failure of cells to undergo immortalization. Therefore, we generated the hTERT-IRES-EGFP construct so as not to affect the function of telomerase to maintain and extend telomeres. CD34 expression by ADSCs decreases over time in culture [27–29]. Therefore, we infected freshly isolated mouse ADSCs containing a highly CD34+ population with retroviruses expressing hTERT-IRES-EGFP genes. Balducci et al. [30] reported that hTERT alone failed to immortalize hADSCs, while hTERT/SV40 or hTERT/HPV E6/E7 co-transductions successfully immortalized cells. In the present study, however, we were able to establish CD34+ mADSCs^hTERT^, suggesting that hTERT alone may immortalize mADSCs and also sustain expression of CD34 on mADSCs. Similarly, Kang et al. [31] also demonstrated that TERT-transduced nonhuman primate ADSCs had an increased level of telomerase activity and increased mean telomere length.

In this study, we found that both CD34+ and CD34- mADSCs^hTERT^ retained their proliferative capacity and multi-potency even after over 100 PDs, irrespective of loss of CD34 expression. Interestingly, CD34+ mADSCs^hTERT^ showed a higher proliferation rate compared to CD34- mADSCs^hTERT^ at 7 and 9 days after seeding, whereas CD34- mADSCs^hTERT^ demonstrated a higher differentiation potential compared to CD34+ mADSCs^hTERT^ (Fig 2). In agreement with our results, a previous study also reported that CD34+ hADSCs were more proliferative and had a greater ability to form colonies compared to CD34- hADSCs, suggesting that CD34 expression in hADSCs may correlate with their replicative potential [29]. In accordance with our observation, Suga et al. [29] also reported that CD34- hADSCs showed a greater ability for differentiation into adipogenic and osteogenic lineages compared to CD34+ counterparts. Taken together, our data and that from a previous report [29] suggest that loss of CD34 expression may be related to the commitment from immature status into mesenchymal lineages.

Primary mADSCs, CD34+, and CD34- mADSCs^hTERT^ primarily expressed EGF, TGF-β1, IGF-1, IGF-2, MCP-1, and HGFR (Fig 3A). VEGF and SDF-1 levels were increased in CD34+ mADSCs^hTERT^ CM compared to CD34- mADSCs^hTERT^ CM (Fig 3B). Previous studies also reported that ADSCs produced a variety of paracrine factors such as VEGF, HGF, and IGF-1 that have pro-angiogenic and/or anti-apoptotic activities [2, 32]. Similarly, hTERT/SV40 and hTERT/HPV E6/E7 demonstrated the capability to secrete significant amounts of VEGF and HGF, showing that immortalized hADSCs maintain a strong capacity to secrete potent angiogenic molecules [30]. The efficacy of ADSC transplantation in the animal model of AMI and hindlimb ischemia via mainly paracrine mechanisms has been demonstrated previously [2–6]. Mazo et al. [33] reported that rat ADSCs improved cardiac function in chronic MI by directly contributing to the vasculature and myofibroblasts and at negligible levels to cardiomyocytes and paracrine activity. Our findings are in line with those of previous studies [33–35], indicating that functional improvement by transplantation of CD34+ and CD34- mADSCs^hTERT^ into infarcted myocardium may be primarily achieved via paracrine actions via cardio-protective, anti-apoptotic effects and angiogenesis rather than direct transdifferentiation.

We observed that CD34+ and CD34- mADSCs^hTERT^ injected into infarcted myocardium resulted in reduction of infarct size and improvement of cardiac function at 28 days after transplantation. No significant difference in regeneration potential between CD34+ and CD34- mADSCs^hTERT^ was found. hADSCs improved postnatal neovascularization in a mouse model of hindlimb ischemia and their effect was correlated with the number of transplanted cells, but not with the ratio of CD34 expression [36]. However, we observed a low engraftment rate as well as rare endothelial differentiation of CD34+ and CD34- mADSCs^hTERT^ at 28 days after transplantation. Therefore, we inferred that low engraftment rate as well as rare endothelial differentiation of transplanted ADSCs may hamper correct evaluation of the functional role of CD34 in regeneration and differentiation potential. In contrast, He et al. [37] reported that differentiation into endothelial cells and participation in blood vessel formation were observed in CD34+ ADSCs, but not in CD34- ADSCs.

Many pro-inflammatory cytokines, TNF-α, IL-1β, IL-6, and MCP-1 were highly induced in experimental models of MI [38–41]. ADSCs have shown anti-inflammatory as well as immunosuppressive activities via secretion of many inflammatory factors [7–9]. In the present study, we found that the secretion levels of IL-6 were severely reduced in CD34+ and CD34- mADSCs^hTERT^ compared to those of primary mADSCs (Fig 3B). In addition, we also found significant decreases in circulating IL-6 levels after transplantation of CD34+ and CD34- mADSCs^hTERT^ groups compared to the AMI control group (Fig 5A). Furthermore, transplantation of CD34- mADSCs^hTERT^ significantly reduced circulating MCP-1 levels compared to the AMI control and CD34+ mADSCs^hTERT^ groups, indicating that strong immunomodulatory action of CD34- mADSCs^hTERT^ may be related to MSC-like characteristics.

Systemic infusion of ADSCs led to a decrease in the level of pro-inflammatory cytokines in multiple animal models [42–46]. hADSCs injected into the flank of mice showed an immunomodulatory function of down-regulating IL-1β and IL-6 [47]. hADSCs also modulated the increases of these pro-inflammatory mediators in serum and in the lung tissue in an endotoxemic rat model [48]. Furthermore, ADSCs CM also significantly reduced levels of the pro-inflammatory cytokines (IL-1β, IL-6, and TNF-α) in the zymosan-induced mouse air pouch model through paracrine mechanisms partly via inhibition of NF-κB activation [49]. Our data and previous reports indicate that the therapeutic effect of ADSCs could be partly attributed to the inhibition of acute inflammatory responses.

In conclusion, we established GFP-tagged CD34+ and CD34- mADSCs^hTERT^ and demonstrated their proliferation capacity, multi-differentiation potential, and secretory profiles *in vitro*. We also demonstrated that transplantation of CD34+ and CD34- mADSCs^hTERT^ into infarcted heart reduced systemic levels of pro-inflammatory cytokines and infarct size, induced neovascularization, and improved cardiac function. Our findings suggest that paracrine factors secreted from transplanted cells may mitigate pro-inflammatory responses and may protect host cardiomyocytes in the infarcted myocardium, contributing to beneficial LV remodeling after MI. However, mechanisms underlying the functional improvement after cell transplantation must be elucidated further. GFP-tagged CD34+ and CD34- mADSCs^hTERT^ are valuable resources for studies on the mechanisms underlying the proliferation, survival, and differentiation of stem cells *in vitro* as well as for the study of cell therapy *in vivo*.

## Supporting Information

S1 Fig Phenotypic characterization of primary mADSCs. (A) Immunofluorescence staining of primary mADSCs shows positive expression for CD34, CD44, CD106, and Sca-1. Nuclei were stained with DAPI. Scale bars = 100 μm. (B) Flow cytometric analysis of primary mADSCs shows positive expression for CD34, CD44, CD106, and Sca-1.
(TIF)

S2 Fig Custom mouse cytokine antibody array. EGF, epidermal growth factor; TGF-β1, transforming growth factor beta 1; HGF, hepatocyte growth factor; IGF-1 and -2, insulin-like growth factor 1 and 2; MCP-1 and -5, monocyte chemotactic protein 1 and 5; VEGF, vascular endothelial growth factor; SDF-1, stromal cell-derived factor 1; bFGF, basic fibroblast growth factor; E-cadherin, epithelial cadherin; HGFR, hepatocyte growth factor receptor; IFN-γ, interferon gamma; IL-10, -6, -1α and -1β, interleukin 10, 6, 1 alpha and 1 beta; IL-1Ra, interleukin 1 receptor antagonist; TNF-α, tumor necrosis factor alpha; CT-1, cardiotrophin 1.
(TIF)

S3 Fig Analysis of changes in body weight and LVEF after CD34+ and CD34- mADSCs^hTERT^ transplantation. (A) No significant differences in body weight changes were found between groups from day 0 to day 28. (B) Delta LVEFs were significantly improved in CD34+ and CD34- mADSCs^hTERT^ groups compared to the control group at day 28 after cell transplantation. Data shown represent mean ± SD (*n* = 10, *p < 0.05; *NS*, not significant). (C) Representative images of FISH staining for Y chromosome showing successful engraftment of the male CD34+ and CD34- mADSCs^hTERT^ in female AMI rats. Arrowheads indicate Y chromosome positive cells. Scale bars = 100 μm.
(TIF)

S4 Fig Transplantation of primary mADSCs and CD34+ and CD34- mADSCs^hTERT^ did not induce tumor formation. (A) No tumor formation was observed in any of the test groups after transplantation of primary mADSCs (*n* = 3), CD34+ (*n* = 3), and CD34- mADSCs^hTERT^ (*n* = 3) into nude mice, as examined up to 6 months after transplantation. (B) No significant differences in body weight changes were found between groups after transplantation of primary mADSCs, CD34+, and CD34- mADSCs^hTERT^ into nude mice. *NS*, not significant.
(TIF)

S5 Fig CD34+ and CD34- mADSCs^hTERT^ have immunomodulatory effects. (A) Coculture experiments to determine rat splenocytes activation by CD34+ or CD34- mADSCs^hTERT^ through costimulatory signals. CD34+ or CD34- mADSCs^hTERT^ were cocultured with rat splenocytes at 1:2, and 1:4 ratios in the presence of PHA and anti-rCD28. Rat splenocyte activation was assessed by determining rIL-2 concentration after 24 h in culture supernatants by ELISA. These results are representative of three independent experiments. (B) The numbers of CD8+ T cells and CD68+ macrophages in the infarcted hearts were quantified using Image-Pro 7.0 software at 72 hrs after CD34+ and CD34- mADSCs^hTERT^ transplantation. Data shown represent mean ± SD (*NS*, not significant) on 8 sections (two fields per section, two sections per heart, *n* = 2 for each group). (C) Representative images showing CD8+ T cells (red), CD68+ macrophages (red) or GFP+ cells (green, arrowheads) in the infarcted hearts at 72 hrs after CD34+ and CD34- mADSCs^hTERT^ transplantation. Boxed regions were magnified in the right panels. Scale bars = 100 μm.
(TIF)

S1 Table Selection of hTERT-immortalized CD34+ and CD34- mADSC lines by limiting dilution. *Cell numbers > 500.
(TIF)

S2 Table Echocardiographic analysis. PWTd, posterior wall thickness in diastole; PWTs, posterior wall thickness in systole; LVEDV, left ventricular end-diastolic volume; LVESV, left ventricular end-systolic volume; EF, ejection fraction. Data shown represent mean ± SD (*n* = 10, *p < 0.05 vs. control).
(TIF)

## References

[1] Varma MJ, Breuls RG, Schouten TE, Jurgens WJ, Bontkes HJ, Schuurhuis GJ, et al Phenotypical and functional characterization of freshly isolated adipose tissue-derived stem cells. Stem Cells Dev. 2007;16(1):91–104. 10.1089/scd.2006.0026 . 17348807

[2] Suzuki E, Fujita D, Takahashi M, Oba S, Nishimatsu H. Adipose tissue-derived stem cells as a therapeutic tool for cardiovascular disease. World J Cardiol. 2015;7(8):454–65. 10.4330/wjc.v7.i8.454 26322185 PMC4549779

[3] Rehman J, Traktuev D, Li J, Merfeld-Clauss S, Temm-Grove CJ, Bovenkerk JE, et al Secretion of angiogenic and antiapoptotic factors by human adipose stromal cells. Circulation. 2004;109(10):1292–8. 10.1161/01.CIR.0000121425.42966.F1 . 14993122

[4] Cai L, Johnstone BH, Cook TG, Liang Z, Traktuev D, Cornetta K, et al Suppression of hepatocyte growth factor production impairs the ability of adipose-derived stem cells to promote ischemic tissue revascularization. Stem Cells. 2007;25(12):3234–43. 10.1634/stemcells.2007-0388 . 17901400

[5] Cho HH, Kim YJ, Kim JT, Song JS, Shin KK, Bae YC, et al The role of chemokines in proangiogenic action induced by human adipose tissue-derived mesenchymal stem cells in the murine model of hindlimb ischemia. Cell Physiol Biochem. 2009;24(5–6):511–8. 10.1159/000257495 . 19910691

[6] Bayes-Genis A, Soler-Botija C, Farre J, Sepulveda P, Raya A, Roura S, et al Human progenitor cells derived from cardiac adipose tissue ameliorate myocardial infarction in rodents. J Mol Cell Cardiol. 2010;49(5):771–80. 10.1016/j.yjmcc.2010.08.010 . 20713059

[7] Banas A, Teratani T, Yamamoto Y, Tokuhara M, Takeshita F, Osaki M, et al IFATS collection: in vivo therapeutic potential of human adipose tissue mesenchymal stem cells after transplantation into mice with liver injury. Stem Cells. 2008;26(10):2705–12. 10.1634/stemcells.2008-0034 . 18535155

[8] DelaRosa O, Lombardo E, Beraza A, Mancheno-Corvo P, Ramirez C, Menta R, et al Requirement of IFN-gamma-mediated indoleamine 2,3-dioxygenase expression in the modulation of lymphocyte proliferation by human adipose-derived stem cells. Tissue Eng Part A. 2009;15(10):2795–806. 10.1089/ten.TEA.2008.0630 . 19231921

[9] Zhang S, Danchuk SD, Bonvillain RW, Xu B, Scruggs BA, Strong AL, et al Interleukin 6 mediates the therapeutic effects of adipose-derived stromal/stem cells in lipopolysaccharide-induced acute lung injury. Stem Cells. 2014;32(6):1616–28. 10.1002/stem.1632 24449042 PMC4365913

[10] Sidney LE, Branch MJ, Dunphy SE, Dua HS, Hopkinson A. Concise review: evidence for CD34 as a common marker for diverse progenitors. Stem Cells. 2014;32(6):1380–9. 10.1002/stem.1661 24497003 PMC4260088

[11] Traktuev DO, Merfeld-Clauss S, Li J, Kolonin M, Arap W, Pasqualini R, et al A population of multipotent CD34-positive adipose stromal cells share pericyte and mesenchymal surface markers, reside in a periendothelial location, and stabilize endothelial networks. Circ Res. 2008;102(1):77–85. 10.1161/CIRCRESAHA.107.159475 . 17967785

[12] Scherberich A, Di Maggio ND, McNagny KM. A familiar stranger: CD34 expression and putative functions in SVF cells of adipose tissue. World J Stem Cells. 2013;5(1):1–8. 10.4252/wjsc.v5.i1.1 23362435 PMC3557347

[13] Miranville A, Heeschen C, Sengenes C, Curat CA, Busse R, Bouloumie A. Improvement of postnatal neovascularization by human adipose tissue-derived stem cells. Circulation. 2004;110(3):349–55. 10.1161/01.CIR.0000135466.16823.D0 . 15238461

[14] Weng NP, Hodes RJ. The role of telomerase expression and telomere length maintenance in human and mouse. J Clin Immunol. 2000;20(4):257–67. . 10939713 10.1023/a:1017223602293

[15] Simonsen JL, Rosada C, Serakinci N, Justesen J, Stenderup K, Rattan SI, et al Telomerase expression extends the proliferative life-span and maintains the osteogenic potential of human bone marrow stromal cells. Nat Biotechnol. 2002;20(6):592–6. 10.1038/nbt0602-592 . 12042863

[16] Zhang X, Soda Y, Takahashi K, Bai Y, Mitsuru A, Igura K, et al Successful immortalization of mesenchymal progenitor cells derived from human placenta and the differentiation abilities of immortalized cells. Biochem Biophys Res Commun. 2006;351(4):853–9. 10.1016/j.bbrc.2006.10.125 . 17094946

[17] Nagai A, Kim WK, Lee HJ, Jeong HS, Kim KS, Hong SH, et al Multilineage potential of stable human mesenchymal stem cell line derived from fetal marrow. PLoS One. 2007;2(12):e1272 18060066 10.1371/journal.pone.0001272 PMC2092394

[18] Anastassiadis K, Rostovskaya M, Lubitz S, Weidlich S, Stewart AF. Precise conditional immortalization of mouse cells using tetracycline-regulated SV40 large T-antigen. Genesis. 2010;48(4):220–32. 10.1002/dvg.20605 . 20146354

[19] Gu Y, Li H, Miki J, Kim KH, Furusato B, Sesterhenn IA, et al Phenotypic characterization of telomerase-immortalized primary non-malignant and malignant tumor-derived human prostate epithelial cell lines. Exp Cell Res. 2006;312(6):831–43. 10.1016/j.yexcr.2005.11.029 . 16413016

[20] Ory DS, Neugeboren BA, Mulligan RC. A stable human-derived packaging cell line for production of high titer retrovirus/vesicular stomatitis virus G pseudotypes. Proc Natl Acad Sci U S A. 1996;93(21):11400–6. 8876147 10.1073/pnas.93.21.11400 PMC38069

[21] Choi YS, Dusting GJ, Stubbs S, Arunothayaraj S, Han XL, Collas P, et al Differentiation of human adipose-derived stem cells into beating cardiomyocytes. J Cell Mol Med. 2010;14(4):878–89. 10.1111/j.1582-4934.2010.01009.x 20070436 PMC3823119

[22] Choi SC, Choi JH, Cui LH, Seo HR, Kim JH, Park CY, et al Mixl1 and Flk1 Are Key Players of Wnt/TGF-beta Signaling During DMSO-Induced Mesodermal Specification in P19 cells. J Cell Physiol. 2015;230(8):1807–21. 10.1002/jcp.24892 . 25521758

[23] Pizzolato MC, Fodor WL. An engineered bifunctional recombinant molecule that regulates humoral and cellular effector functions of the immune system. Transplantation. 2003;75(4):542–9. 10.1097/01.TP.0000048492.91165.FC . 12605124

[24] Thompson CB, Lindsten T, Ledbetter JA, Kunkel SL, Young HA, Emerson SG, et al CD28 activation pathway regulates the production of multiple T-cell-derived lymphokines/cytokines. Proc Natl Acad Sci U S A. 1989;86(4):1333–7. 2465550 10.1073/pnas.86.4.1333 PMC286684

[25] Dredge K, Marriott JB, Todryk SM, Muller GW, Chen R, Stirling DI, et al Protective antitumor immunity induced by a costimulatory thalidomide analog in conjunction with whole tumor cell vaccination is mediated by increased Th1-type immunity. J Immunol. 2002;168(10):4914–9. . 11994441 10.4049/jimmunol.168.10.4914

[26] Counter CM, Hahn WC, Wei W, Caddle SD, Beijersbergen RL, Lansdorp PM, et al Dissociation among in vitro telomerase activity, telomere maintenance, and cellular immortalization. Proc Natl Acad Sci U S A. 1998;95(25):14723–8. 9843956 10.1073/pnas.95.25.14723 PMC24516

[27] Yoshimura K, Shigeura T, Matsumoto D, Sato T, Takaki Y, Aiba-Kojima E, et al Characterization of freshly isolated and cultured cells derived from the fatty and fluid portions of liposuction aspirates. J Cell Physiol. 2006;208(1):64–76. 10.1002/jcp.20636 . 16557516

[28] Mitchell JB, McIntosh K, Zvonic S, Garrett S, Floyd ZE, Kloster A, et al Immunophenotype of human adipose-derived cells: temporal changes in stromal-associated and stem cell-associated markers. Stem Cells. 2006;24(2):376–85. 10.1634/stemcells.2005-0234 . 16322640

[29] Suga H, Matsumoto D, Eto H, Inoue K, Aoi N, Kato H, et al Functional implications of CD34 expression in human adipose-derived stem/progenitor cells. Stem Cells Dev. 2009;18(8):1201–10. 10.1089/scd.2009.0003 . 19226222

[30] Balducci L, Blasi A, Saldarelli M, Soleti A, Pessina A, Bonomi A, et al Immortalization of human adipose-derived stromal cells: production of cell lines with high growth rate, mesenchymal marker expression and capability to secrete high levels of angiogenic factors. Stem Cell Res Ther. 2014;5(3):63 10.1186/scrt452 24887516 PMC4055112

[31] Kang SK, Putnam L, Dufour J, Ylostalo J, Jung JS, Bunnell BA. Expression of telomerase extends the lifespan and enhances osteogenic differentiation of adipose tissue-derived stromal cells. Stem Cells. 2004;22(7):1356–72. . 15579653 10.1634/stemcells.2004-0023

[32] Nakanishi C, Nagaya N, Ohnishi S, Yamahara K, Takabatake S, Konno T, et al Gene and protein expression analysis of mesenchymal stem cells derived from rat adipose tissue and bone marrow. Circ J. 2011;75(9):2260–8. . 21747191 10.1253/circj.cj-11-0246

[33] Mazo M, Cemborain A, Gavira JJ, Abizanda G, Arana M, Casado M, et al Adipose stromal vascular fraction improves cardiac function in chronic myocardial infarction through differentiation and paracrine activity. Cell Transplant. 2012;21(5):1023–37. 10.3727/096368911X623862 . 22305117

[34] Ranganath SH, Levy O, Inamdar MS, Karp JM. Harnessing the mesenchymal stem cell secretome for the treatment of cardiovascular disease. Cell Stem Cell. 2012;10(3):244–58. 10.1016/j.stem.2012.02.005 22385653 PMC3294273

[35] Li N, Wang C, Jia L, Du J. Heart regeneration, stem cells, and cytokines. Regen Med Res. 2014;2(1):6 10.1186/2050-490X-2-6 25984334 PMC4390097

[36] Moon MH, Kim SY, Kim YJ, Kim SJ, Lee JB, Bae YC, et al Human adipose tissue-derived mesenchymal stem cells improve postnatal neovascularization in a mouse model of hindlimb ischemia. Cell Physiol Biochem. 2006;17(5–6):279–90. 10.1159/000094140 . 16791003

[37] He J, Duan H, Xiong Y, Zhang W, Zhou G, Cao Y, et al Participation of CD34-enriched mouse adipose cells in hair morphogenesis. Mol Med Rep. 2013;7(4):1111–6. 10.3892/mmr.2013.1307 . 23404453

[38] Frangogiannis NG, Lindsey ML, Michael LH, Youker KA, Bressler RB, Mendoza LH, et al Resident cardiac mast cells degranulate and release preformed TNF-alpha, initiating the cytokine cascade in experimental canine myocardial ischemia/reperfusion. Circulation. 1998;98(7):699–710. . 9715863 10.1161/01.cir.98.7.699

[39] Frangogiannis NG, Youker KA, Rossen RD, Gwechenberger M, Lindsey MH, Mendoza LH, et al Cytokines and the microcirculation in ischemia and reperfusion. J Mol Cell Cardiol. 1998;30(12):2567–76. 10.1006/jmcc.1998.0829 . 9990529

[40] Dewald O, Ren G, Duerr GD, Zoerlein M, Klemm C, Gersch C, et al Of mice and dogs: species-specific differences in the inflammatory response following myocardial infarction. Am J Pathol. 2004;164(2):665–77. 10.1016/S0002-9440(10)63154-9 14742270 PMC1602262

[41] Vandervelde S, van Luyn MJ, Rozenbaum MH, Petersen AH, Tio RA, Harmsen MC. Stem cell-related cardiac gene expression early after murine myocardial infarction. Cardiovasc Res. 2007;73(4):783–93. 10.1016/j.cardiores.2006.11.030 . 17208206

[42] Kim JM, Lee ST, Chu K, Jung KH, Song EC, Kim SJ, et al Systemic transplantation of human adipose stem cells attenuated cerebral inflammation and degeneration in a hemorrhagic stroke model. Brain Res. 2007;1183:43–50. 10.1016/j.brainres.2007.09.005 . 17920570

[43] Gonzalez MA, Gonzalez-Rey E, Rico L, Buscher D, Delgado M. Adipose-derived mesenchymal stem cells alleviate experimental colitis by inhibiting inflammatory and autoimmune responses. Gastroenterology. 2009;136(3):978–89. 10.1053/j.gastro.2008.11.041 . 19135996

[44] Sun CK, Chang CL, Lin YC, Kao YH, Chang LT, Yen CH, et al Systemic administration of autologous adipose-derived mesenchymal stem cells alleviates hepatic ischemia-reperfusion injury in rats. Crit Care Med. 2012;40(4):1279–90. 10.1097/CCM.0b013e31823dae23 . 22336724

[45] Lee SC, Kim JO, Kim SJ. Secretome from human adipose-derived stem cells protects mouse liver from hepatic ischemia-reperfusion injury. Surgery. 2015;157(5):934–43. 10.1016/j.surg.2014.12.016 . 25704431

[46] Mert T, Kurt AH, Arslan M, Celik A, Tugtag B, Akkurt A. Anti-inflammatory and Anti-nociceptive Actions of Systemically or Locally Treated Adipose-Derived Mesenchymal Stem Cells in Experimental Inflammatory Model. Inflammation. 2015;38(3):1302–10. 10.1007/s10753-014-0101-1 . 25563206

[47] Dong Z, Peng Z, Chang Q, Lu F. The survival condition and immunoregulatory function of adipose stromal vascular fraction (SVF) in the early stage of nonvascularized adipose transplantation. PLoS One. 2013;8(11):e80364 10.1371/journal.pone.0080364 24260375 PMC3832367

[48] Shin S, Kim Y, Jeong S, Hong S, Kim I, Lee W, et al The therapeutic effect of human adult stem cells derived from adipose tissue in endotoxemic rat model. Int J Med Sci. 2013;10(1):8–18. 10.7150/ijms.5385 23289000 PMC3534872

[49] Carceller MC, Guillen MI, Ferrandiz ML, Alcaraz MJ. Paracrine in vivo inhibitory effects of adipose tissue-derived mesenchymal stromal cells in the early stages of the acute inflammatory response. Cytotherapy. 2015;17(9):1230–9. 10.1016/j.jcyt.2015.06.001 . 26276006

