# Supplementary figures and images for: Transplantation of Immortalized CD34+ and CD34- Adipose-Derived Stem Cells Improve Cardiac Function and Mitigate Systemic Pro-Inflammatory Responses
Source: PLoS One. 2016 Feb 3;11(2):e0147853. doi: 10.1371/journal.pone.0147853 (PMC4740491; doi:10.1371/journal.pone.0147853)

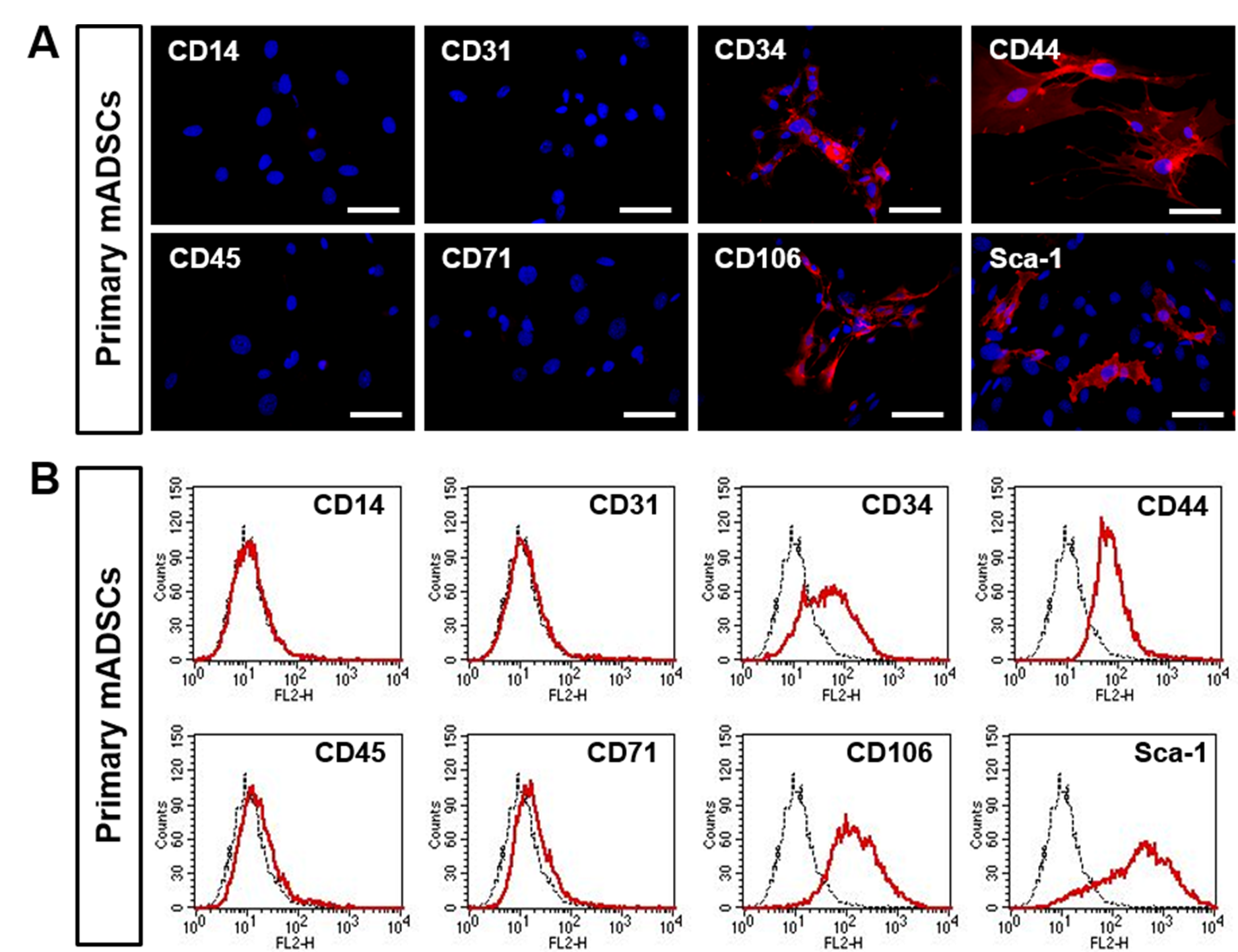

Supplement: S1 Fig — (A) Immunofluorescence staining of primary mADSCs shows positive expression for CD34, CD44, CD106, and Sca-1. Nuclei were stained with DAPI. Scale bars = 100 μm. (B) Flow cytometric analysis of primary mADSCs shows positive expression for CD34, CD44, CD106, and Sca-1. (TIF) [file pone.0147853.s001.tif]

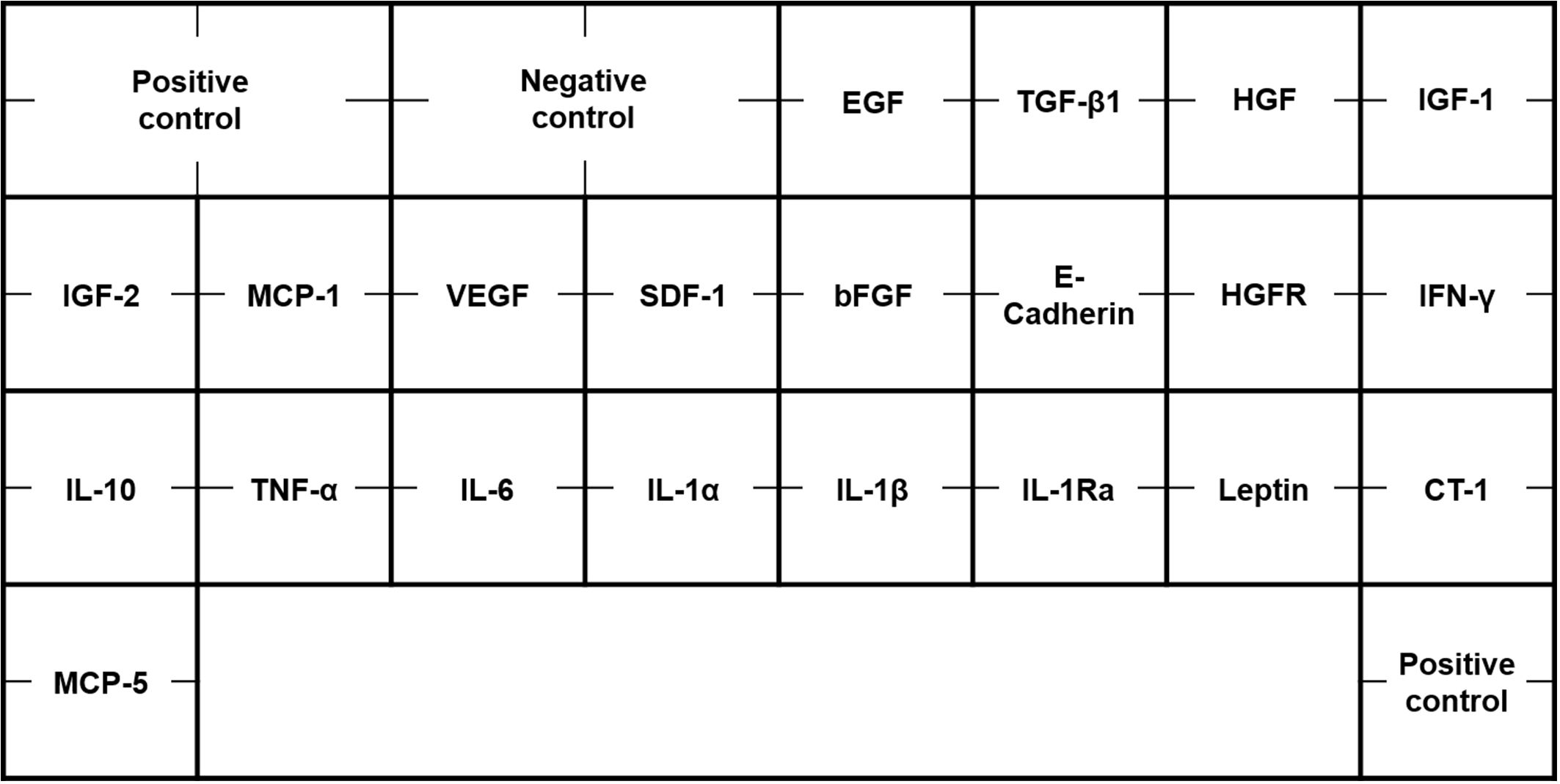

Supplement: S2 Fig — EGF, epidermal growth factor; TGF-β1, transforming growth factor beta 1; HGF, hepatocyte growth factor; IGF-1 and -2, insulin-like growth factor 1 and 2; MCP-1 and -5, monocyte chemotactic protein 1 and 5; VEGF, vascular endothelial growth factor; SDF-1, stromal cell-derived factor 1; bFGF, basic fibroblast growth factor; E-cadherin, epithelial cadherin; HGFR, hepatocyte growth factor receptor; IFN-γ, interferon gamma; IL-10, -6, -1α and -1β, interleukin 10, 6, 1 alpha and 1 beta; IL-1Ra, interleukin 1 receptor antagonist; TNF-α, tumor necrosis factor alpha; CT-1, cardiotrophin 1. (TIF) [file pone.0147853.s002.tif]

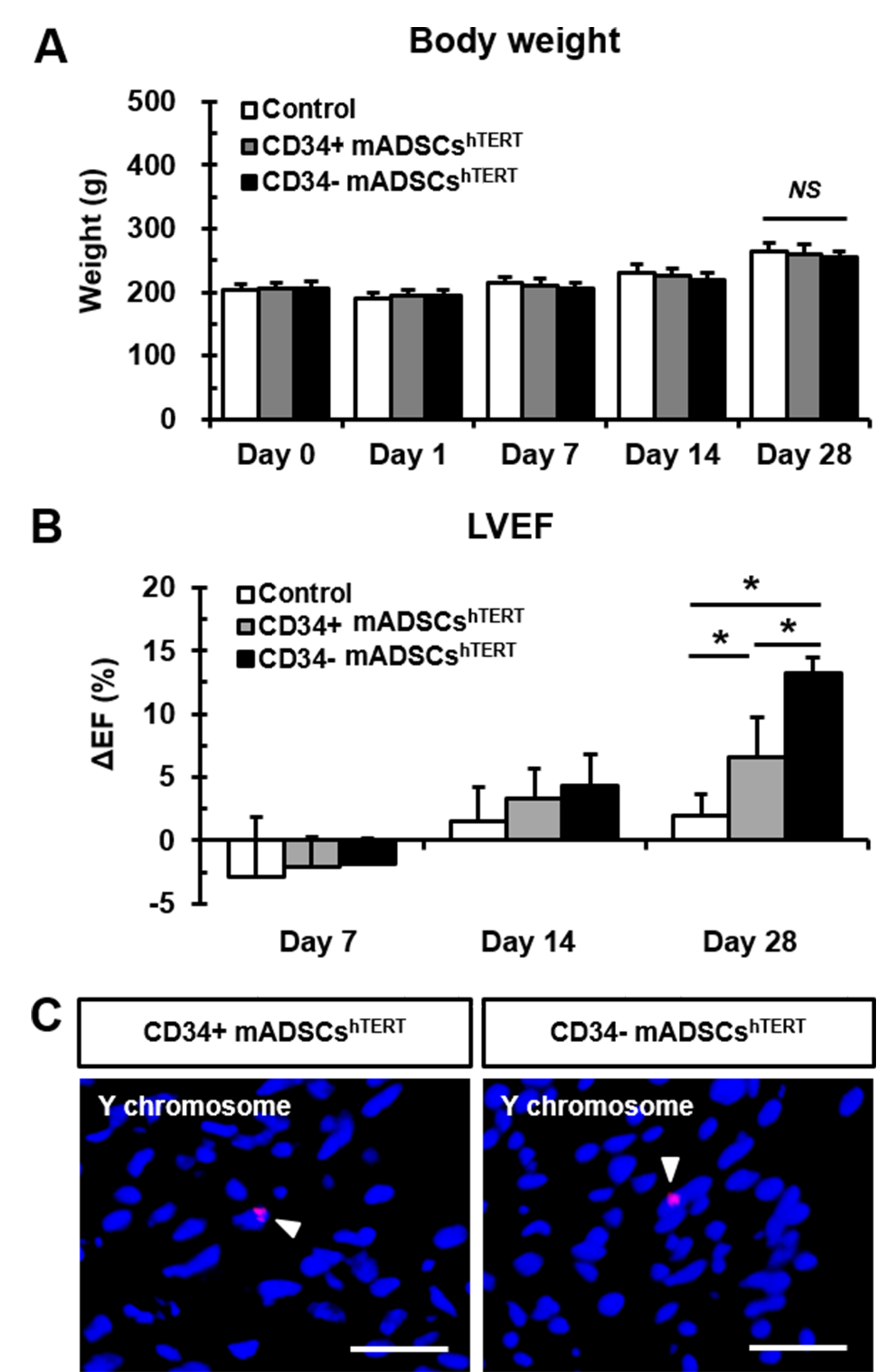

Supplement: S3 Fig — (A) No significant differences in body weight changes were found between groups from day 0 to day 28. (B) Delta LVEFs were significantly improved in CD34+ and CD34- mADSCshTERT groups compared to the control group at day 28 after cell transplantation. Data shown represent mean ± SD (n = 10, *p < 0.05; NS, not significant). (C) Representative images of FISH staining for Y chromosome showing successful engraftment of the male CD34+ and CD34- mADSCshTERT in female AMI rats. Arrowheads indicate Y chromosome positive cells. Scale bars = 100 μm. (TIF) [file pone.0147853.s003.tif]

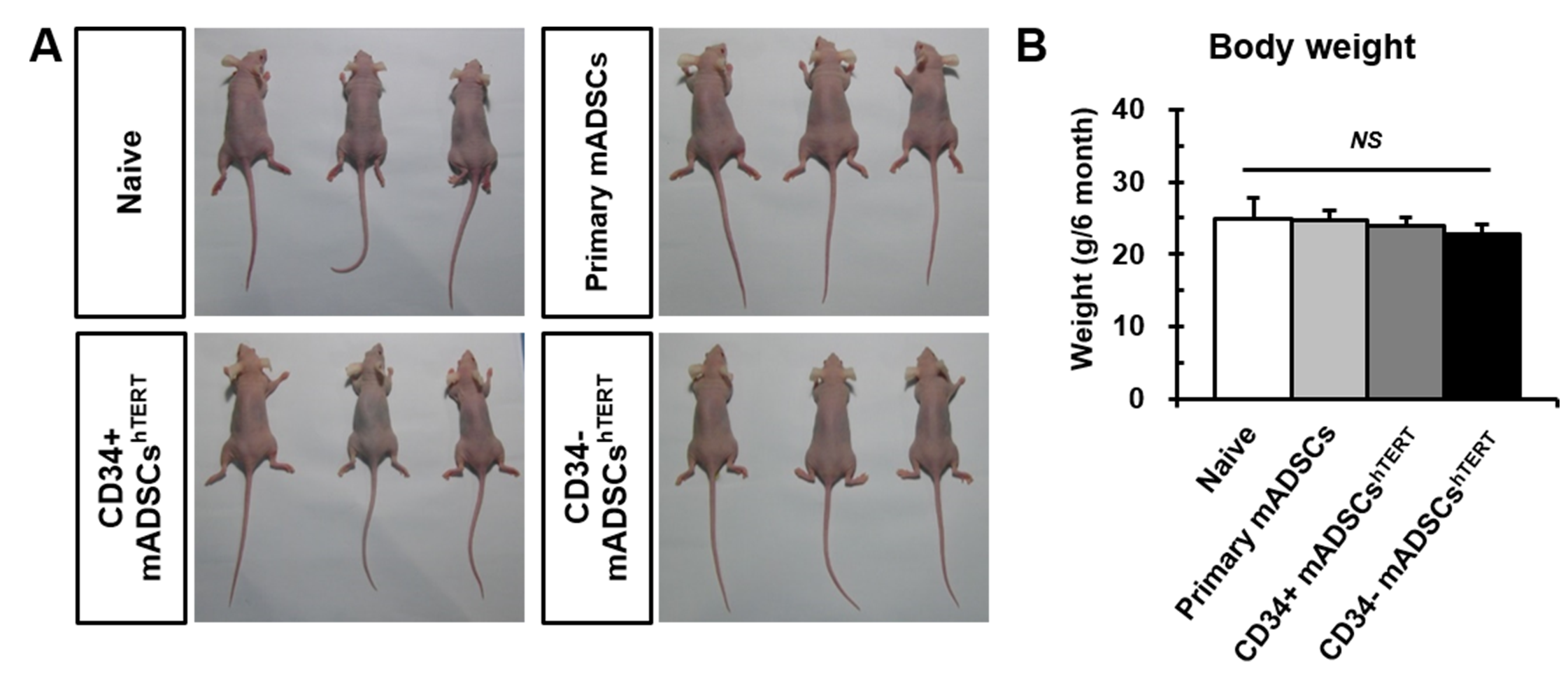

Supplement: S4 Fig — (A) No tumor formation was observed in any of the test groups after transplantation of primary mADSCs (n = 3), CD34+ (n = 3), and CD34- mADSCshTERT (n = 3) into nude mice, as examined up to 6 months after transplantation. (B) No significant differences in body weight changes were found between groups after transplantation of primary mADSCs, CD34+, and CD34- mADSCshTERT into nude mice. NS, not significant. (TIF) [file pone.0147853.s004.tif]

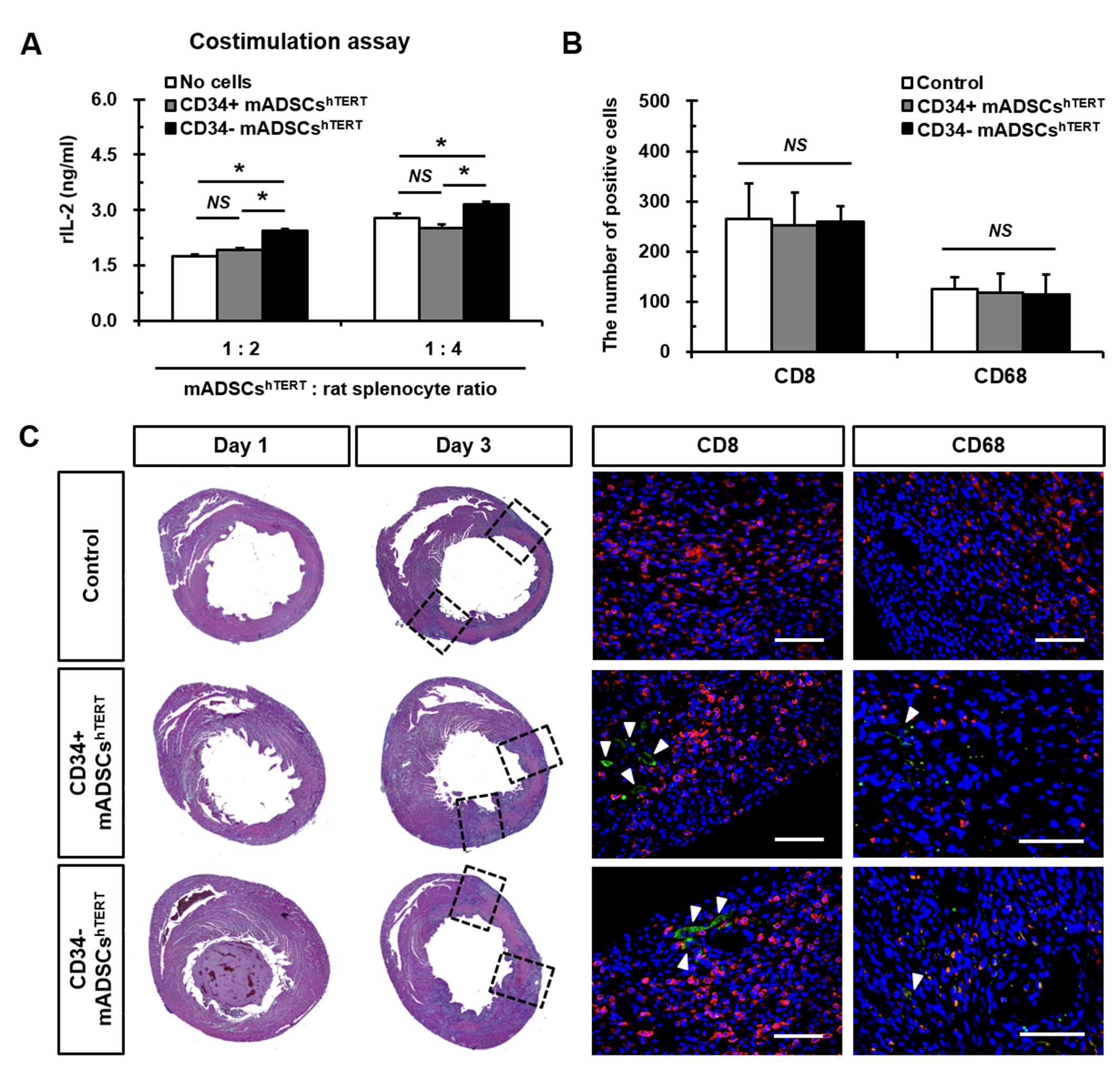

Supplement: S5 Fig — (A) Coculture experiments to determine rat splenocytes activation by CD34+ or CD34- mADSCshTERT through costimulatory signals. CD34+ or CD34- mADSCshTERT were cocultured with rat splenocytes at 1:2, and 1:4 ratios in the presence of PHA and anti-rCD28. Rat splenocyte activation was assessed by determining rIL-2 concentration after 24 h in culture supernatants by ELISA. These results are representative of three independent experiments. (B) The numbers of CD8+ T cells and CD68+ macrophages in the infarcted hearts were quantified using Image-Pro 7.0 software at 72 hrs after CD34+ and CD34- mADSCshTERT transplantation. Data shown represent mean ± SD (NS, not significant) on 8 sections (two fields per section, two sections per heart, n = 2 for each group). (C) Representative images showing CD8+ T cells (red), CD68+ macrophages (red) or GFP+ cells (green, arrowheads) in the infarcted hearts at 72 hrs after CD34+ and CD34- mADSCshTERT transplantation. Boxed regions were magnified in the right panels. Scale bars = 100 μm. (TIF) [file pone.0147853.s005.tif]

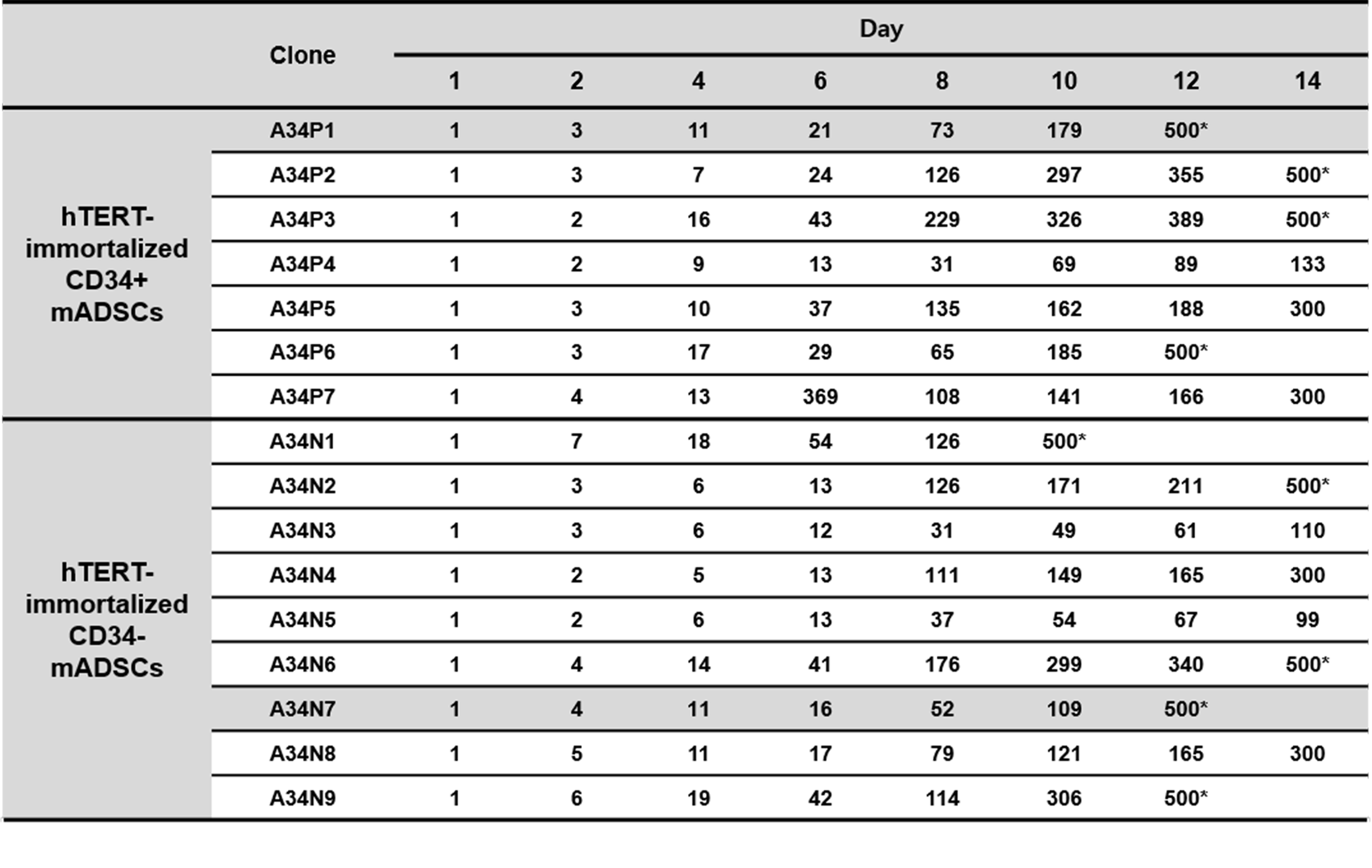

Supplement: S1 Table — *Cell numbers > 500. (TIF) [file pone.0147853.s006.tif]

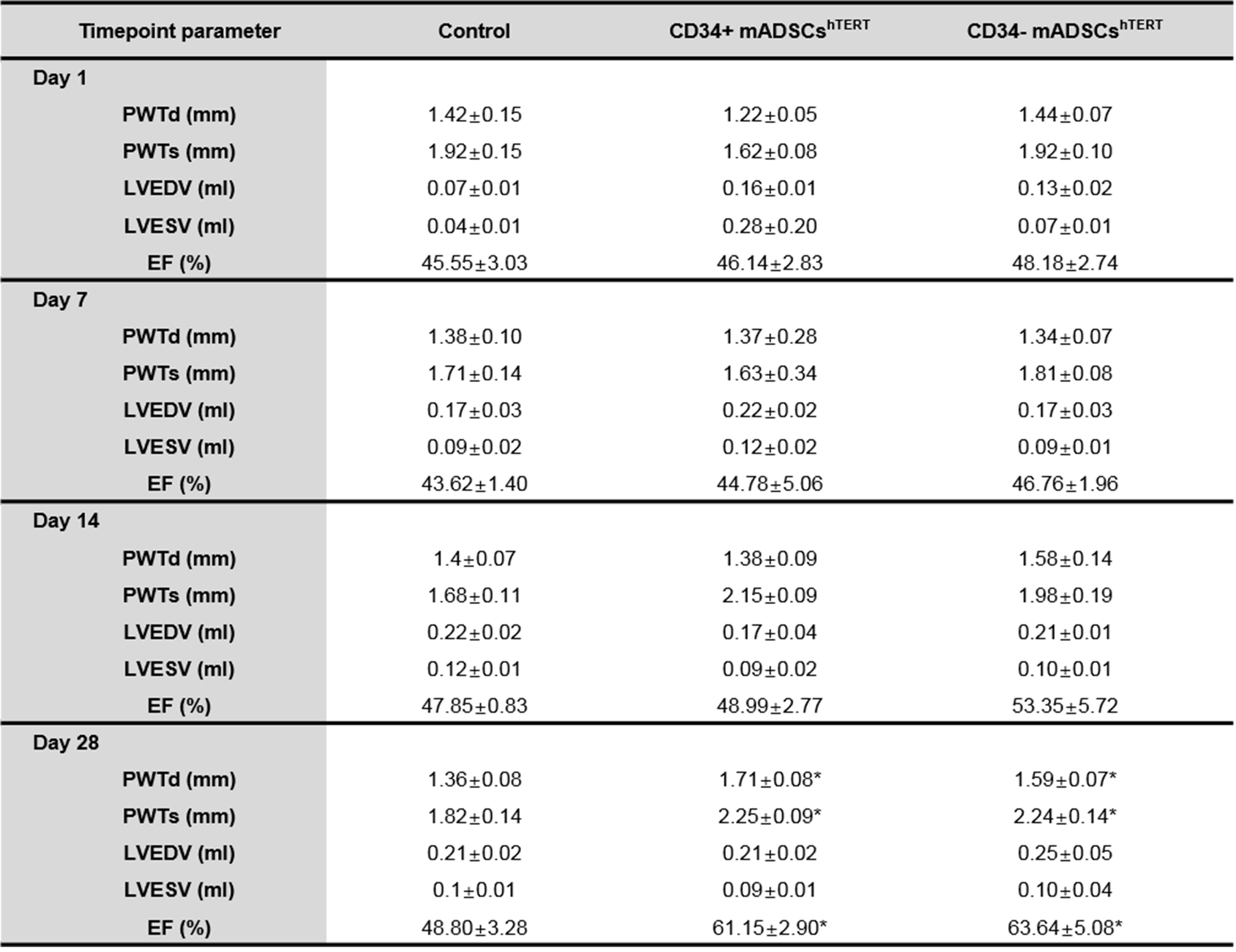

Supplement: S2 Table — PWTd, posterior wall thickness in diastole; PWTs, posterior wall thickness in systole; LVEDV, left ventricular end-diastolic volume; LVESV, left ventricular end-systolic volume; EF, ejection fraction. Data shown represent mean ± SD (n = 10, *p < 0.05 vs. control). (TIF) [file pone.0147853.s007.tif]
